# Insights into plant cell wall structure, architecture, and integrity using glycome profiling of native and AFEX^TM^-pre-treated biomass

**DOI:** 10.1093/jxb/erv107

**Published:** 2015-04-23

**Authors:** Sivakumar Pattathil, Michael G. Hahn, Bruce E. Dale, Shishir P. S. Chundawat

**Affiliations:** ^1^Complex Carbohydrate Research Center, University of Georgia, Athens, GA 30602, USA; ^2^BioEnergy Science Center, Oak Ridge National Laboratory, Oak Ridge, TN 37831, USA; ^3^DOE Great Lakes Bioenergy Research Center, Department of Chemical Engineering and Materials Science, Michigan State University, East Lansing, MI 48824, USA; ^4^ Present address: Department of Chemical and Biochemical Engineering, C-150A Engineering Building, Rutgers The State University of New Jersey, Piscataway, NJ 08854, USA

**Keywords:** AFEX, biofuels, cell walls, glycome profiling, plant biomass, recalcitrance.

## Abstract

Cell walls, which constitute the bulk of plant biomass, vary considerably in their structure, composition, and architecture. Studies on plant cell walls can be conducted on both native and pre-treated plant biomass samples, allowing an enhanced understanding of these structural and compositional variations. Here glycome profiling was employed to determine the relative abundance of matrix polysaccharides in several phylogenetically distinct native and pre-treated plant biomasses. Eight distinct biomass types belonging to four different subgroups (i.e. monocot grasses, woody dicots, herbaceous dicots, and softwoods) were subjected to various regimes of AFEX™ (ammonia fiber expansion) pre-treatment [AFEX is a trademark of MBI, Lansing (http://www.mbi.org]. This approach allowed detailed analysis of close to 200 cell wall glycan epitopes and their relative extractability using a high-throughput platform. In general, irrespective of the phylogenetic origin, AFEX™ pre-treatment appeared to cause loosening and improved accessibility of various xylan epitope subclasses in most plant biomass materials studied. For most biomass types analysed, such loosening was also evident for other major non-cellulosic components including subclasses of pectin and xyloglucan epitopes. The studies also demonstrate that AFEX™ pre-treatment significantly reduced cell wall recalcitrance among diverse phylogenies (except softwoods) by inducing structural modifications to polysaccharides that were not detectable by conventional gross composition analyses. It was found that monitoring changes in cell wall glycan compositions and their relative extractability for untreated and pre-treated plant biomass can provide an improved understanding of variations in structure and composition of plant cell walls and delineate the role(s) of matrix polysaccharides in cell wall recalcitrance.

## Highlight

Glycome profiling and AFEX pre-treatment reveal differences in cell wall architecture among different plant taxa and implicate subclasses of polysaccharides whose loosening results in reduced recalcitrance of walls to deconstruction.

## Introduction

Plant cell walls vary considerably in their structure and composition depending on a number of factors, including the species of origin, tissue type, stage of development, and environmental conditions (Freshour *et al.*, 1996; Knox, 2008; Pauly and Keegstra, 2008; Popper, 2008). These variations in physico-chemical and/or ultrastructural features of cell walls are largely governed by the relative composition and proportion of various cell wall components that include (but are not limited to) cellulose, lignin, hemicelluloses, and pectic polysaccharides, as well as the interactions between these polymers. Cell walls are innately resistant to deconstruction by biological or chemical catalysts, and this property is often referred to as ‘recalcitrance’ (Himmel *et al.*, 2007). Cell wall recalcitrance is considered as the primary barrier to cost-effective cellulosic biofuel production (Himmel *et al.*, 2007; Chundawat *et al.*, 2011*a*). Thus, it is imperative to obtain a better understanding of plant cell wall structure and architecture in order to develop rational approaches to overcome the cell wall recalcitrance barrier.

The cell walls of herbaceous dicots, woody dicots, monocot grasses, and woody gymnosperms, plant groups that include most species being targeted as candidate biomass feedstocks for sustainable biofuel production, have clearly distinguishable compositions, as demonstrated by chemical analyses (Pauly and Keegstra, 2008; Vogel, 2008). For instance, the walls of commelinoid monocots (e.g. the grasses), in comparison with those of dicots, non-commelinoid monocots, and gymnosperms, mainly differ in the relative abundances of non-cellulosic polysaccharides and their associations/linkages (Carpita and Gibeaut, 1993; Carpita, 1996; Vogel, 2008). Primary walls in grasses are characterized by the significant abundance of the hemicellulosic polysaccharides, glucuronoarabinoxylans and mixed-linkage glucans, with relatively minor proportions of xyloglucans, pectic polysaccharides, and structural proteins such as arabinogalactan proteins. In contrast, dicot and gymnosperm primary walls, in general, are characterized by a greater abundance of xyloglucans in the former, and mannans and glucomannans in the latter as the predominant hemicelluloses, and relatively higher abundances of pectic polysaccharides and structural proteins (Vogel, 2008). Secondary walls vary considerably in structure and composition from primary walls, typically containing a higher proportion of cellulose and less pectic polysaccharides, with the predominant non-cellulosic components in secondary cell walls of grasses and woody dicots being xylans and lignin, with glucomannans also present to a small extent in woody dicots (Vogel, 2008). Lignin and hemicelluloses are also the major non-cellulosic components of secondary walls in woody gymnosperms; however, galactomannan and glucomannan are more prominent hemicelluloses in these walls in addition to xylan (Aspinall, 1980; Capek *et al.*, 2002; Pauly and Keegstra, 2008). Lignin compositions, in general, also vary among plant families (Boerjan *et al.*, 2003). Woody dicot and monocot grass lignins contain predominantly guaiacyl (G) and syringyl (S) units with traces of hydroxyphenyl (H) units; the grasses typically contain higher levels of H units. In contrast, woody gymnosperm lignins are primarily composed of G units with lower amounts of H units.

Information about structural and compositional features of plant cell walls that has been obtained so far has emerged primarily from chemical analyses of intact cell walls or of individual polymers that were extracted/isolated from those cell walls. Comparative studies of plant cell walls from diverse species have been challenging, given the structural complexity, compositional heterogeneity, and diversity of cell walls originating from diverse plant species, especially when the number of samples to be analysed is large.

The recent development of a large and diverse library of plant glycan-directed monoclonal antibodies (mAbs) (Pattathil *et al.*, 2010) has made possible new antibody-based approaches to plant cell wall characterization (Moller *et al.*, 2007; Knox, 2008). One antibody-based approach that is particularly suitable for the analysis of large numbers of samples is glycome profiling (Moller *et al.*, 2007, 2008; Pattathil *et al.*, 2012*a*). Glycome profiling involves preparation of a set of sequential cell wall extracts followed by their screening with a comprehensive suite of cell wall glycan-directed mAbs. The sequential extraction steps employ increasingly harsh chemical treatments, which result in the fractionation of non-cellulosic matrix polysaccharides based on how tightly the polysaccharides are integrated into the cell wall. These extractive chemical reagents disrupt the interactions/associations among cell wall components, facilitating their release as soluble glycan-rich extractives that can be readily screened in diverse experimental platforms (Moller *et al.*, 2007; Pattathil *et al.*, 2012*a*; Pattathil *et al*., 2012*b*; Demartini *et al.*, 2013). In the authors’ laboratory, an enzyme-linked immunosorbent assay (ELISA)-based screen is employed using a large and diverse suite of cell wall glycan-directed mAbs (Pattathil *et al.*, 2010) that enables semi-quantitative detection of most major non-cellulosic glycans in the cell wall extracts. This approach has been applied to diverse cell wall studies, including genetically modified plants (Shen *et al.*, 2013; Tan *et al.*, 2013; Zhao *et al.*, 2013), microbially modified plant biomass (Chung *et al.*, 2014; Izquierdo *et al.*, 2014), and pre-treated plant biomass (Demartini *et al.*, 2011, 2013; Li *et al.*, 2014; Socha *et al.*, 2014) that have yielded new insights into the linkages/associations that exist among cell wall components.

Other recently developed approaches for the study of plant cell walls have arisen out of an increasing interest in the use of plant biomass, which consists largely of cell walls, as a sustainable feedstock for biofuel production. These studies have presented new methods for altering overall cell wall structure and composition to reduce its recalcitrance to deconstruction (Mood *et al.*, 2013). One such method is AFEX™ (ammonia fiber expansion) (Chundawat *et al.*, 2011*b*; Campbell *et al.*, 2013), which is a physico-chemical pre-treatment that can reduce biomass recalcitrance to enzymatic deconstruction with varying efficiency depending on pre-treatment severity and the plant biomass being treated (Chundawat *et al.*, 2011*b*, 2013). Recent work has revealed that AFEX™ pre-treatment can cause cleavage of key lignin–carbohydrate ester linkages, followed by dissolution, extraction, and deposition of cell wall extractives on outer plant cell wall surfaces (Chundawat *et al.*, 2011*b*). This results in the creation of a nano-porous network within plant cell walls that enhances matrix polysaccharide accessibility to hydrolytic enzymes (Chundawat *et al.*, 2011*b*; Ciesielski *et al.*, 2013).

In the current study, AFEX™ pre-treatment was employed to alter the composition and architecture of cell walls from eight plant species originating from diverse phylogenetic classes and then the altered walls were characterized using glycome profiling to identify changes in the walls that lead to reduced recalcitrance and hence draw inferences about overall wall architecture in the various plants tested.

## Materials and methods

### Biomass source and AFEX™ pre-treatment

Poplar (*Populus nigra*) was provided by the National Renewable Energy Laboratory (Golden, CO, USA) and processed as described elsewhere (Balan *et al.*, 2009*a*). Goldenrod [*Solidago* sp; 2010 harvest from Michigan State University, Kellogg Biological Station (MSU KBS)], corn stover (*Zea mays*; 2008 harvest), and switchgrass (*Panicum virgatum*; 2009 harvest) were received from Great Lakes Bioenergy Research Center (GLBRC) at Michigan State University (MSU). Black locust (*Robinia pseudoacacia*) was harvested at MSU in 2008 and processed as described previously (Garlock *et al.*, 2012). Loblolly pine wood (*Pinus taeda*) harvested from the southeastern USA was a kind gift from Professor Art Ragauskas (Georgia Institute of Technology). Douglas fir wood (*Pseudotsuga menziesii*) harvested from northwestern Canada was a kind gift from Professor John Saddler (University of British Columbia). Alfalfa hay (*Medicago sativa*) was harvested from MSU farms in 2011 and was procured from the GLBRC at MSU. All biomass samples were dried to <20% moisture (dry weight basis) and milled with a Wiley mill using a 1mm size mesh screen. Milled biomass samples were pre-treated using the AFEX™ protocol as described elsewhere (Balan *et al.*, 2009*b*; Chundawat *et al.*, 2010). Briefly, samples were AFEX™ treated for three pre-treatment conditions, namely: (i) low severity (low): 0.5:1 ammonia to dry biomass loading, 60% moisture loading, 70 °C for 5min total treatment time; (ii) medium severity (medium): 1.5:1 ammonia to dry biomass loading, 60% moisture loading, 100 °C for 30min total treatment time; and (iii) high severity (severe): 5:1 ammonia to dry biomass loading, 5% moisture loading, 130 °C for 120min total treatment time. All pre-treated samples and appropriate controls were stored at 4 °C prior to glycome profiling.

### Glycome profiling of biomass residues

Sequential cell wall extractions and glycome profiling of various biomass residues were carried out as described previously (Demartini *et al.*, 2011; Pattathil *et al.*, 2012*a*). Plant glycan-directed mAbs (Pattathil *et al.*, 2010) were from laboratory stocks (CCRC, JIM, and MAC series) at the Complex Carbohydrate Research Center (available through CarboSource Services; http://www.carbosource.net) or were obtained from BioSupplies (Australia) (BG1, LAMP). A description of the mAbs used in this study can be found in Supplementary Table S1 available at *JXB* online, which includes links to a web database, Wall*MAb*DB (http://www.wallmabdb.net) that provides detailed information about each antibody.

Scatter plots were generated using normalized epitope abundance values per unit mass of the original biomass/cell walls calculated using the following equation (Li *et al.*, 2014):

$$\left[\left({\text{OD}}_{\text{45}0}–{\text{OD}}_{\text{655}}\right){\text{ mg}}^{–\text{1}}\text{original biomass}\right]={\text{N}}_{1}\times {\text{N}}_{2}\times {\text{N}}_{3}\times {\text{N}}_{4}$$

where, N_1_=[(OD_450_–OD_655_) μg^–1^ glucose equivalent], N_2_=[(μg glucose equivalent) mg^–1^ extracted material], N_3_=[(mg extracted material) g^–1^ original biomass], and N_4_=[(1g)/(1000mg)].

Note that in scatter plot visualizations, an apparent enhancement in the normalized epitope abundance in individual extracts from pre-treated (medium AFEX™ regime) biomass compared with untreated biomass will appear to the left of the *x=y* diagonal line and an apparent decrease in the epitope abundance in an extract due to pre-treatment will cause a shift to the right (Li *et al.*, 2014).

### Biomass polysaccharide and lignin composition analysis

The untreated biomass polysaccharide composition was estimated at the GLBRC Cell Wall Analytical Facility (http://www.glbrc.org) based on a previously published protocol (Foster *et al.*, 2010*a*). Briefly, biomass was ball milled with the GLBRC iWall robotic system and extracted with water and then alcohol to prepare alcohol-insoluble residue (AIR). AIR biomass was incubated with amylase and pullulanase and washed with water to remove starch. The isolated residue was dried and weighed into three technical replicates (2mg dry weight each) to determine gross polysaccharide composition. Cell wall matrix polysaccharides (rhamnose, fucose, arabinose, xylose, mannose, galactose, and glucose) were estimated after acid hydrolysis and solubilization by 2M trifluoroacetic acid (TFA) and further quantification by gas chromatography–mass spectrometry (GC-MS) as corresponding alditol acetates derivatives. The crystalline cellulose residue isolated after TFA hydrolysis was hydrolysed in 72% sulphuric acid and assayed using a colorimetric anthrone method, as described previously (Foster *et al.*, 2010*a*).

Untreated biomass total lignin content and composition was estimated at the GLBRC Cell Wall Analytical Facility (http://www.glbrc.org) based on a previously published protocol (Foster *et al.*, 2010*b*). Briefly, the AIR biomass residue was treated with freshly made acetyl bromide solution [25% (v/v) acetyl bromide in glacial acetic acid) at 50 °C for 2h and treated on ice with 2M sodium hydroxide and 0.5M hydroxylamine hydrochloride. The resulting solution was aliquoted into a UV plate and read in an ELISA reader at 280nm. The percentage acetyl bromide-solubilized lignin (ABSL) was estimated as described elsewhere (Foster *et al.*, 2010*b*). The coefficients used to estimate ABSL were 18.21 (hardwoods), 17.75 (monocot grasses), 13.785 (softwoods), and 15.297 (herbaceous dicots). A detailed protocol for estimating relative syringyl (S), guaiacyl (G), and hydroxyphenyl (H) lignin composition of untreated biomass samples based on a modified thioacidolysis, trimethylsilyl (TMS) derivatization and GC-MS analysis method is provided elsewhere (Foster *et al.*, 2010*b*).

### Biomass enzymatic saccharification

Untreated and pre-treated biomass samples were hydrolysed by a commercial cellulase cocktail (C.Tec2 from Novozymes; 193mg ml^–1^ total protein concentration) at a glucan loading of 1% (w/v), in 50mM citrate buffer (pH 5.0) in a 15ml total reaction volume. The samples were hydrolysed at 50 °C with gentle agitation (90rpm) for a total duration of 168h. Total cellulase loading employed for biomass saccharification was 15mg total protein g^–1^ glucan. Supernatants were removed at 24h and 168h, filtered, and analysed for monomeric sugars (glucose, xylose) by high-performance liquid chromatography (HPLC) as described previously (Chundawat *et al.*, 2010).

## Results and discussion

The eight phylogenetically distinct plant biomass employed in this study belong to four different subgroups of plants [i.e. monocot grasses, woody dicots (hardwoods), herbaceous dicots, and woody gymnosperms (softwoods)], enabling studies on broad and structurally variant cell walls constituting the biomass of a diversity of plants of interest as feedstocks for sustainable biofuel production. The overall goal of the current study is to use glycome profiling to understand the existing variations in structural, compositional, and linkage/association among wall components across diverse phylogenies. The studies also analysed AFEX-pre-treated cell walls to gain further insight into how walls are put together by examining how AFEX treatments alter linkages/associations among wall components. These studies take advantage of the effect of AFEX pre-treatment regimes on overall wall structure to reduce the recalcitrance of plant biomass to deconstruction, thereby providing useful insights into structural features of plant cell walls that underlie recalcitrance.

The structural integrity of plant cell walls, which most probably causes cell wall recalcitrance, is the net result of physico-chemical features of the various wall polymers and the complex interactions/associations among those components. Wall components that contribute to the structural integrity of cell walls include (but are not limited to) cellulose, lignin, hemicellulose, and pectin, (Zhao *et al.*, 2012). For example, non-covalent interactions such as hydrogen bonding and van der Waals forces are primarily responsible for the integrity of cellulose microfibrils (O’Sullivan, 1997) and the cellulose–hemicellulose associations that are thought to form one of the major polymer networks within plant cell walls (Ding *et al.*, 2012; Silveira *et al.*, 2013). Covalent cross-links and ionic interactions play major roles in the formation and integrity of pectic matrices, another of the major polymer networks within the wall (Iiyama *et al.*, 1994; Mohnen, 2008). Lignin–carbohydrate complexes that are held together via ester and ether linkages between lignin and hemicelluloses can further strengthen wall integrity (Iiyama *et al.*, 1994; Boukari *et al.*, 2009; Achyuthan *et al.*, 2010). Among diverse cell walls, the strengths of the associations/interactions between various cell wall components, especially the interactions of non-cellulosic components such as hemicelluloses with the cellulosic core and lignin, are subject to significant variation. For example, in grasses, esterification of ferulic acids to arabinoxylans leads to the formation of ferulate-based polysaccharide–lignin complexes that contribute to biomass recalcitrance (Buanafina, 2009). Other examples in dicots include the diverse interactions of hemicelluloses [e.g. xyloglucans, (glucurono)xylans, and glucomannans] with cellulose driven by variations in the strengths of hydrogen bonding owing to their diverse structures (reviewed by Ong *et al.*, 2014). Additionally, recent studies using *Arabidopsis* have speculated on the relevance of glucuronic acid and acetyl substituents for governing xylan interactions with cellulose and lignin (Bromley *et al.*, 2013). In summary, many variations exist among cell walls originating from diverse phylogenies that affect their integrity and hence their recalcitrance to deconstruction.

### Glycome profiling of biomass residues before and after AFEX™ pre-treatment regimes

The comprehensive collection of plant cell wall glycan-directed antibodies that was used in this study is broad enough to monitor cell wall epitope composition contributed by most major non-cellulosic glycan components of the walls. The glycome profiling data sets obtained by screening of the cell wall extracts obtained from the eight plant species examined in this study were analysed by two approaches: (i) comparative analyses of heat map representations of the raw data from all untreated and pre-treated biomass samples; and (ii) analyses of scatter plots generated using normalized epitope abundance values per unit mass of the original cell walls from untreated and medium severity AFEX™-pre-treated biomass samples. There are ongoing efforts to develop novel ammonia pre-treatments at higher ammonia loadings that allow conversion of native cellulose I to more readily digestible cellulose III allomorphs (Chundawat *et al.*, 2011d). However, currently only medium regime AFEX™-pre-treated samples were considered for scatter plot analyses, as these represented the optimal AFEX™ regime (based on ammonia loading) that is relevant to industrial biomass applications (Wang *et al.*, 1998; Eggeman and Elander, 2005; Bals *et al.*, 2011; Campbell *et al.*, 2013; Chundawat *et al.*, 2013).

**Fig. 1.**
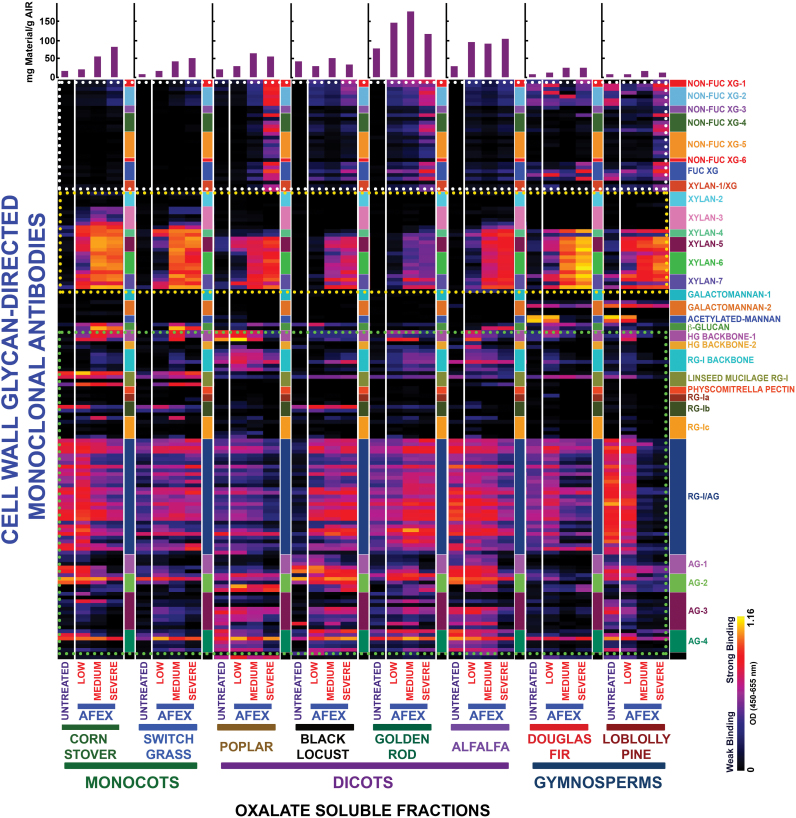
Heat map analyses of the relative abundance of major non-cellulosic cell wall glycan epitopes in oxalate extracts from eight phylogenetically diverse plant biomasses with or without AFEX™ pre-treatment. Oxalate extracts were prepared from cell walls isolated from diverse classes of plant biomass as explained in the Materials and Methods. The extracts were subsequently screened by ELISA using a comprehensive suite of cell wall glycan-directed mAbs. Binding response values are depicted as heat maps with a black–red–bright yellow colour scheme, where bright yellow represents the strongest binding and black no binding. The dotted boxes outline sets of antibodies whose binding signals were used for the scatter plot analyses shown in Fig. 2. The amount of carbohydrate material recovered per gram of cell wall is depicted in the bar graphs (purple) above the heat maps. The panel on the right-hand side of the heat map shows the groups of mAbs based on the class of cell wall glycan they each recognize.

**Fig. 2.**
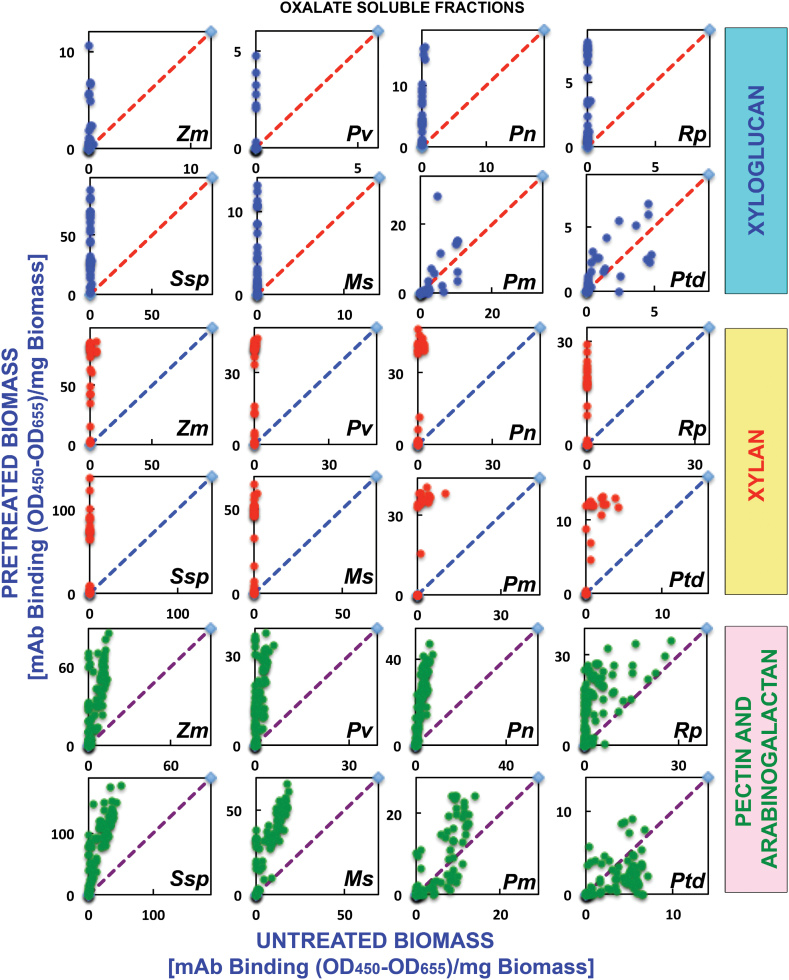
Scatter plot analyses of the relative abundance of major non-cellulosic cell wall glycan epitopes in oxalate extracts from eight phylogenetically diverse plant biomasses with or without AFEX™ pre-treatment. Oxalate extracts were prepared from cell walls isolated from diverse classes of plant biomass as explained in the Materials and Methods. The extracts were subsequently screened by ELISA using a comprehensive suite of cell wall glycan-directed mAbs. Comparisons of the relative abundances of epitopes characteristic of three cell wall polysaccharide classes, xyloglucans (blue dots), xylans (red dots), and pectin/arabinogalactans (green dots), in the oxalate extracts before and after medium severity AFEX™ pre-treatment of diverse plant biomass samples, corn stover (*Zm*), switchgrass (*Pv*), poplar (*Pn*), black locust (*Rp*), golden rod (*Ssp*), alfalfa (*Ms*), Douglas fir (*Pm*), and loblolly pine (*Ptd*). Data are re-plotted from Fig. 1, but are normalized to represent mAb binding strength per mass of original cell wall. The red dashed lines denote the expected position if the abundance of these glycan epitopes was unchanged after AFEX™ pre-treatment. Data points above and below the dashed lines represent increased or decreased glycan epitope abundance, respectively, after AFEX™ pre-treatment. Note that the *y*-axis scales are different for individual plots to permit visualization of trends and magnitudes of normalized epitope abundances.

The glycome profiling data are organized by cell wall extract for untreated biomass and biomass pre-treated under three regimes of AFEX™, namely low, medium, and severe (see the Materials and Methods for details) for all eight plant species analysed. Examination of the heat maps resulting from the antibody screening of the oxalate-solubilized material (first extraction stage) (Fig. 1) shows that increasing AFEX™ pre-treatment severity significantly enhanced the presence of epitopes recognized by the xylan-4 to xylan-7 groups of mAbs (that can recognize both unsubstituted homoxylans and substituted xylans such as glucuronoxylans or arabinoxylans; Schmidt *et al*., 2015) for all plant species examined (Fig. 1; yellow dotted block). In the case of monocot grasses, a similar enhancement in the oxalate extracts was also observed for epitopes recognized by the xylan-3 group of mAbs. Scatter plot analyses of the glycome profiling data (Fig. 2) substantiated these conclusions as an enhanced abundance of xylan epitopes was observed in the oxalate extracts in all medium AFEX™ severity pre-treated biomass samples compared with untreated controls. For other biomass types, enhanced binding of xyloglucan-directed mAbs to the oxalate extracts from the most severe AFEX™ regimes was evident (Fig. 1; white dotted block). Supporting this observation, scatter plot analyses for biomass samples pre-treated with the medium AFEX™ severity regime indicated enhanced extractability of xyloglucan epitopes in all cases except for the two gymnosperms, Douglas fir and loblolly pine, where no obvious trends were apparent. These results suggest that AFEX™ pre-treatment leads to enhanced extractability of xyloglucan epitopes only in biomass materials of angiosperm origin. Scatter plot analyses of the pectin and arabinogalactan epitopes (those recognized by the homogalacturonan- and rhamnogalacturonan-I-backbone groups and the rhamnogalacturonan-I/arabinogalactan and various arabinogalactan groups of mAbs, respectively) present within the oxalate extracts exhibited similar trends to the case of xyloglucan epitopes, with enhanced abundance of all of these epitopes in oxalate extracts of pre-treated samples for all biomasses except the two gymnosperms, which showed both increased and decreased extractabilities of pectin and arabinogalactan epitopes as a result of AFEX™ pre-treatment; loblolly pine, in particular, showed a large number of arabinogalactan epitopes with reduced extractability by oxalate (Fig. 2), especially for the most severe pre-treatment regime (Fig. 1). In general, oxalate extracted more material from the walls of monocots and dicots than from the walls of gymnosperms (Fig. 1, bar graphs); the herbaceous dicot biomasses yielded the most oxalate-extractable material.

**Fig. 3.**
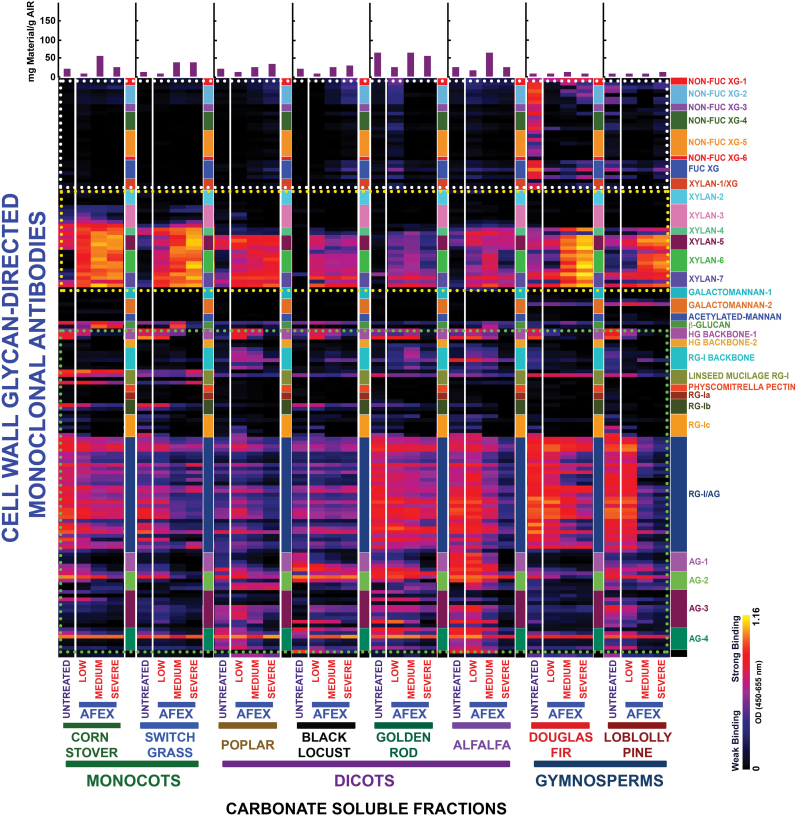
Heat map analyses of the relative abundance of major non-cellulosic cell wall glycan epitopes in carbonate extracts from eight phylogenetically diverse plant biomasses with or without AFEX™ pre-treatment. Carbonate extracts were prepared from cell walls isolated from diverse classes of plant biomass as explained in the Materials and Methods. The extracts were subsequently screened by ELISA using a comprehensive suite of cell wall glycan-directed mAbs. Binding response values are depicted as heat maps with a black–red–bright yellow colour scheme, where bright yellow represents the strongest binding and black no binding. The dotted boxes outline sets of antibodies whose binding signals were used for the scatter plot analyses shown in Fig. 4. The amount of carbohydrate material recovered per gram of cell wall is depicted in the bar graphs (purple) above the heat maps. The panel on the right-hand side of the heat map shows the groups of mAbs based on the class of cell wall glycan they each recognize.

**Fig. 4.**
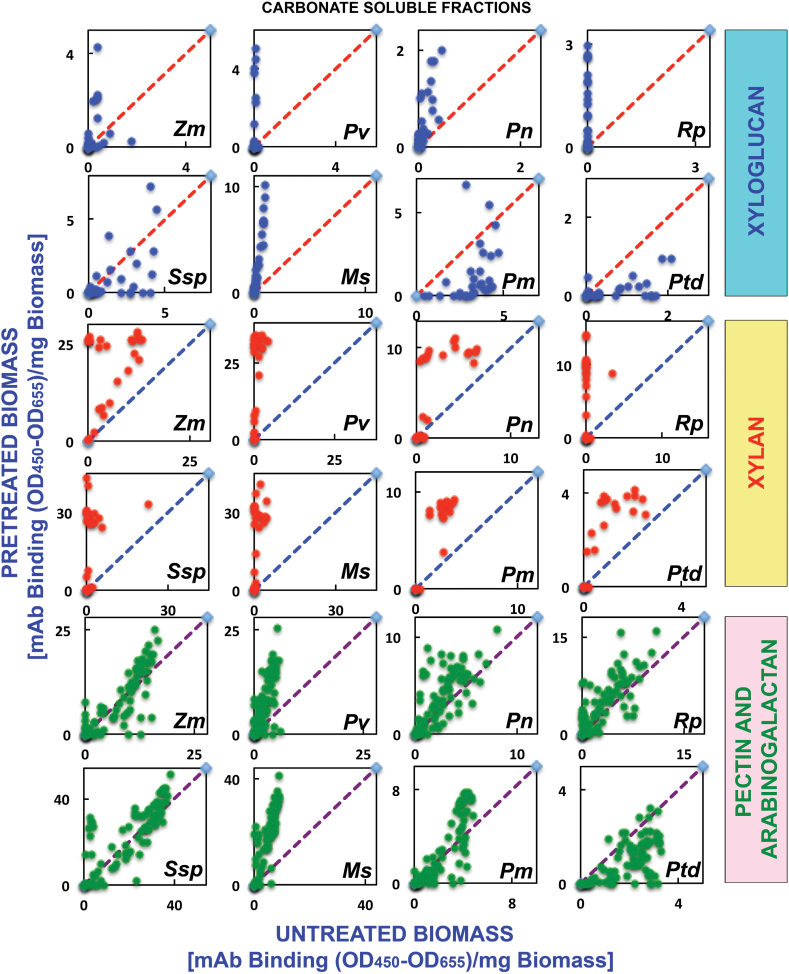
Scatter plot analyses of the relative abundance of major non-cellulosic cell wall glycan epitopes in carbonate extracts from eight phylogenetically diverse plant biomasses with or without AFEX™ pre-treatment. Carbonate extracts were prepared from cell walls isolated from diverse classes of plant biomass as explained in the Materials and Methods. The extracts were subsequently screened by ELISA using a comprehensive suite of cell wall glycan-directed mAbs. Comparisons of the relative abundances of epitopes characteristic of three cell wall polysaccharide classes, xyloglucans (blue dots), xylans (red dots), and pectin/arabinogalactans (green dots), in the carbonate extracts before and after medium severity AFEX™ pre-treatment of diverse plant biomass samples (see Fig. 2 for more details). Data are re-plotted from Fig. 3, but are normalized to represent mAb binding strength per mass of original cell wall. The red dashed lines denote the expected position if the abundance of these glycan epitopes was unchanged after AFEX™ pre-treatment. Data points above and below the dashed lines represent increased or decreased glycan epitope abundance, respectively, after AFEX™ pre-treatment. Note that the *y*-axis scales are different for individual plots to permit visualization of trends and magnitudes of normalized epitope abundances.

The trends observed in heat maps of the oxalate extracts were continued in the heat maps of the carbonate extracts (second extraction stage); that is, the carbonate extracts exhibited an enhanced abundance of various xylan epitopes (Fig. 3, yellow dotted blocks) after AFEX™ pre-treatment compared with untreated biomass. Again, scatter plot analyses substantiated these results, with increased abundance of xylan epitopes in all pre-treated biomass samples (Fig. 4). Closer inspection of both oxalate and carbonate heat maps revealed that some xylan epitope groups [e.g. xylan-6 mAbs, which recognize unsubstituted homoxylan epitope structures (Schmidt *et al.*, 2015)] are more recalcitrant and require harsher AFEX™ pre-treatment conditions to facilitate their removal compared with other xylan epitope groups (e.g. xylan-4, -5, and -7). AFEX™ pre-treatment also resulted in enhanced extractability of xyloglucan epitopes for all of the biomass samples with the exception of golden rod, a herbaceous dicot, and both gymnosperms. The abundance of pectin and arabinogalactan epitopes in carbonate extracts from pre-treated biomass as depicted by scatter plots showed little enhanced extractability of these epitopes after AFEX™ pre-treatment for all biomass samples except for switchgrass and alfalfa, which showed increased abundance of almost all of these epitopes (Fig. 4).

Some apparent discrepancies were observed between the heat maps and the scatter plots of the oxalate and carbonate extracts, particularly for the pectin and arabinogalactan epitopes. In general, the scatter plots showed an enhanced extractability of the majority of pectin and arabinogalacatan epitopes from all biomass types (except loblolly pine). However, this is not concomitantly reflected in heat maps of the raw data for these extracts. These apparent discrepancies could arise from two sources. First, there is a greatly enhanced proportion of hemicellulosic glycans, particularly xylans, in the oxalate and carbonate extracts after AFEX™ pre-treatment. Since samples are loaded onto the ELISA plates on an equal sugar basis, a reduced binding intensity of pectin- and arabinogalactan-directed mAbs would be expected due to their reduced relative proportion in the extracts. Secondly, the formula used to calculate the scatter plots includes the total sugar released. However, smaller glycans are not reproducibly immobilized on the ELISA plates. If the medium AFEX™ conditions induce fragmentation of pectins and/or arabinogalactans, then a lower proportion of these epitopes would be detected in the ELISA screen, resulting in apparent lower signal intensities on the heat maps.

**Fig. 5.**
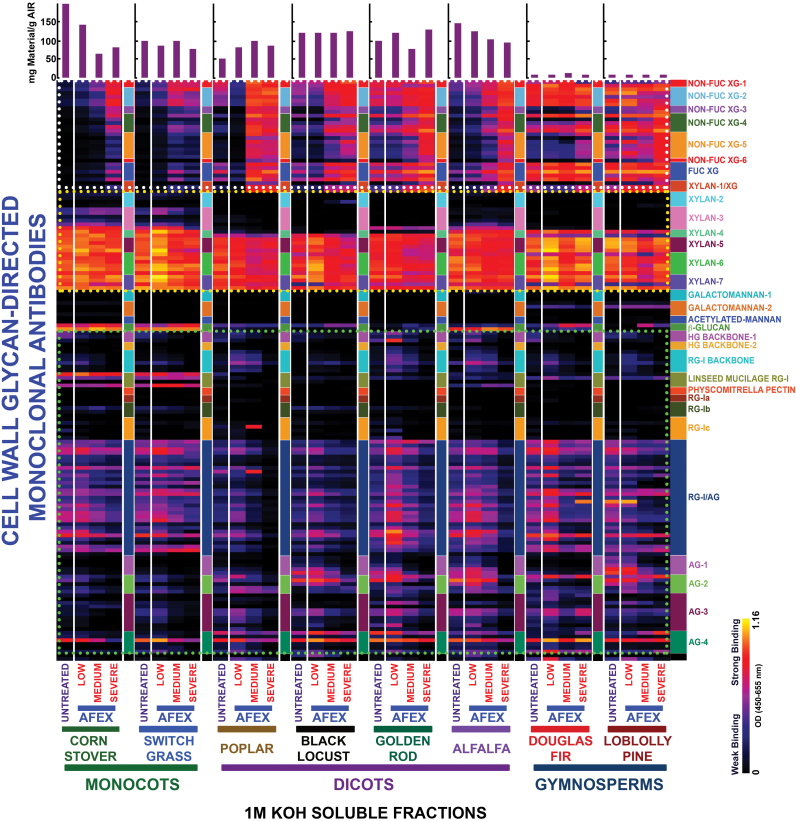
Heat map analyses of the relative abundance of major non-cellulosic cell wall glycan epitopes in 1M KOH extracts from eight phylogenetically diverse plant biomasses with or without AFEX™ pre-treatment. The 1M KOH extracts were prepared from cell walls isolated from diverse classes of plant biomass as explained in the Materials and Methods. The extracts were subsequently screened by ELISA using a comprehensive suite of cell wall glycan-directed mAbs. Binding response values are depicted as heat maps with a black–red–bright yellow colour scheme, where bright yellow represents the strongest binding and black no binding. The dotted boxes outline sets of antibodies whose binding signals were used for the scatter plot analyses shown in Fig. 6. The amount of carbohydrate material recovered per gram of cell wall is depicted in the bar graphs (purple) above the heat maps. The panel on the right-hand side of the heat map shows the groups of mAbs based on the class of cell wall glycan they each recognize.

**Fig. 6.**
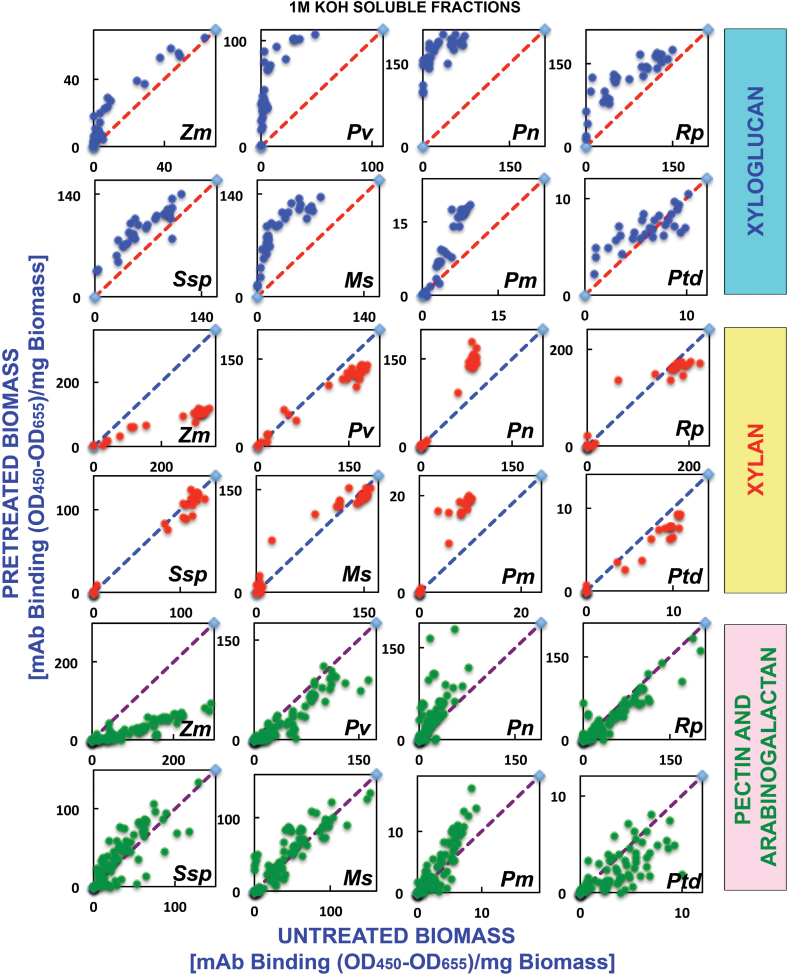
Scatter plot analyses of the relative abundance of major non-cellulosic cell wall glycan epitopes in 1M KOH extracts from eight phylogenetically diverse plant biomasses with or without AFEX™ pre-treatment. The 1M KOH extracts were prepared from cell walls isolated from diverse classes of plant biomass as explained in the Materials and Methods. The extracts were subsequently screened by ELISA using a comprehensive suite of cell wall glycan-directed mAbs. Comparisons of the relative abundances of epitopes characteristic of three cell wall polysaccharide classes, xyloglucans (blue dots), xylans (red dots), and pectin/arabinogalactans (green dots), in the 1 M KOH extracts before and after medium severity AFEX™ pre-treatment of diverse plant biomass samples (see Fig. 2 for more details). Data are re-plotted from Fig. 5, but are normalized to represent mAb binding strength per mass of original cell wall. The red dashed lines denote the expected position if the abundance of these glycan epitopes was unchanged after AFEX™ pre-treatment. Data points above and below the dashed lines represent increased or decreased glycan epitope abundance, respectively, after AFEX™ pre-treatment. Note that the *y*-axis scales are different for individual plots to permit visualization of trends and magnitudes of normalized epitope abundances.

Extraction of both native and pre-treated biomass with 1M KOH (third extraction step) yielded dramatically less material for the two gymnosperm biomasses compared with the other samples (Fig. 5, bar graphs). The most significant difference observed in the glycome profiles of the 1M KOH extracts was the enhanced extractability of xyloglucan epitopes from all pre-treated biomass samples, irrespective of their phylogenetic origin. This was particularly true for the medium and severely pre-treated samples as observed on the heat maps (Fig. 5; white dotted blocks), as well as in the scatter plot analyses of the medium severity AFEX™ pre-treatment data (Fig. 6). For loblolly pine, enhanced extractability of fewer xyloglucan epitopes after AFEX™ pre-treatment was observed compared with the other biomass samples. In general, AFEX™ pre-treatment appeared to have little effect on glycome profiles of the base-extractable xylan and pectin/arabinogalactan epitopes when comparing pre-treated with untreated biomass samples. This is most easily seen in the scatter plots (Fig. 6), where most of the points fall along or close to the diagonal *x=y* line. Interestingly, AFEX™-pre-treated corn stover showed lower abundance of both xylan and pectin/arabinogalactan epitopes versus untreated biomass, probably due to the fact that a significant fraction of these matrix polymers had been removed from the pre-treated samples in the oxalate and carbonate extractions.

In general, far fewer differences between untreated and AFEX™-pre-treated biomass samples were noted in the glycome profiles of the three harshest extractives, 4M KOH (fourth extraction stage), chlorite (fifth extraction stage), and 4M KOH PC (sixth extraction stage). The heat maps obtained from the raw data of mAb screening of materials solubilized by these three extraction reagents were mostly similar between untreated and pre-treated biomass, as demonstrated by visual comparisons of mAb binding intensities (Supplementary Figs S1, S3, S5 at *JXB* online). However, in order to see whether any discernible and meaningful trends existed that might correlate with the phylogenetic diversity of the plant biomass types employed in this study, further analyses of these extracts were mainly focused on the scatter plots for the medium severity AFEX™ pre-treatment (Supplementary Figs S2, S4, S6) derived from the respective glycome profiling data sets. Given the complexity of these data sets, the analyses were made simpler by generating a table (Supplementary Table S2) that depicts the overall qualitative abundance of epitopes comprising xyloglucan, xylan, and pectin/arabinogalactan components in extracts from pre-treated biomass in comparison with untreated biomass. Epitope abundances were depicted as ‘enhanced’ (scatter plot data that show a complete shift to the right of the ‘*x*=*y*’ line), ‘reduced’ (scatter plot data that show a complete shift to the left of the ‘*x*=*y*’ line), or ‘no change’ (scatter plot data showing no apparent shift from the ‘*x*=*y*’ line). General conclusions drawn from these glycome profiling data sets suggest that pre-treated biomass of the two herbaceous dicots, golden rod and alfalfa, exhibited similar trends in epitope abundance in these extracts (Supplementary Table S2, light green highlights). For instance, a higher abundance of xyloglucan epitopes was noted in the 4M KOH extracts of pre-treated golden rod and alfalfa biomasses, while reduced abundances of xyloglucan, xylan, and pectin/arabinogalactan epitopes were observed in the chlorite and 4M KOHPC extracts in these two pre-treated biomasses. The two gymnosperm biomasses (Douglas fir and loblolly pine) also exhibited similar trends in the materials released by the three harshest extractive reagents. Both pre-treated gymnosperms exhibited increased xyloglucan epitope abundance in the 4M KOHPC extract and increased xylan epitope abundance in both chlorite and 4M KOHPC extracts. These results suggest that delignification of AFEX™-pre-treated gymnosperms improved hemicellulose extractability. Pre-treated corn stover biomass showed reduced abundance of all cell wall glycan epitopes, which suggested that these epitopes are probably removed under less harsh extraction conditions. In switchgrass, similarities with corn stover were observed only in the cases of reduced abundances of xyloglucan epitopes in chlorite and pectin/arabinogalactan epitopes in chlorite and 4M KOHPC extracts from pre-treated biomass. In a similar way, in the case of the woody angiosperm dicots (poplar and black locust), a different pattern was observed, with pre-treated poplar biomass exhibiting reduced abundance of all cell wall glycan epitopes analysed, except in the case of pectin/arabinogalactan epitopes in 4M KOH. Finally, black locust exhibited enhanced glycan epitope abundance in pre-treated biomass in most cases. Overall, the analyses of glycome profiling data on the three most harsh severity treatment extracts from untreated and pre-treated biomass from diverse phylogenetic classes again displayed the complexity and diversity of cell wall architecture and demonstrated that AFEX™ pre-treatment causes varying modifications on plant cell walls to reduce biomass recalcitrance.

### Variations in biomass composition and enzymatic hydrolysis of untreated and AFEX™-pre-treated plant biomass

Biomass sugar and lignin content/composition for all eight untreated samples are provided in Supplementary Figs S7 and S8 at *JXB* online. As expected, the cellulose content of the monocot grasses was higher (~37–38%; dry weight basis) than for the herbaceous dicots (~26–31%), but marginally lower than for the two woody dicots (~38–40%) and softwoods (~40–41%). For the two Poales, arabinoxylans were the major hemicelluloses, as indicated by the high content of xylose (~27%) and arabinose (~3.5–4%), but lower content of other hemicellulosic sugars (such as mannose and galactose). Similarly, the dicot biomass had a high content of xylose (~12–16%) and arabinose (~1–4%). In contrast, the softwoods had a higher mannose (~9–12%) and galactose (~2%) content compared with xylose (~4–6%), indicating that the predominant hemicellulosic polymers were galactomannans for these feedstocks.

Lignin compositions, as determined by acetyl bromide solubility analyses (Supplementary Fig. S8A at *JXB* online), indicated that softwoods had a significantly higher lignin content (~34–38%) compared with both monocots (~20–24%) and dicots (~15–20%). More detailed characterization of lignin monomer compositions (syringyl or S units, guaiacyl or G units, and *p*-hydroxyphenyl or H units) for the eight biomass samples (Supplementary Fig. S8B) indicated that the two monocot grasses contained predominantly SG-type lignin, with S and G subunits at comparable levels and with a significant fraction of H units (~2.2–3.4%; on a mass basis). The two woody dicots and goldenrod also contained predominantly SG-type lignin with a lower fraction of H units (~0.3–0.5%) and with an S/G ratio ranging between 1.9 and 2.5. The herbaceous dicot, alfalfa, had a lignin composition that was more similar to that of monocots than to that of other dicots. Finally, the softwoods were composed almost exclusively of G units with trace amounts of S and H units.

**Fig. 7.**
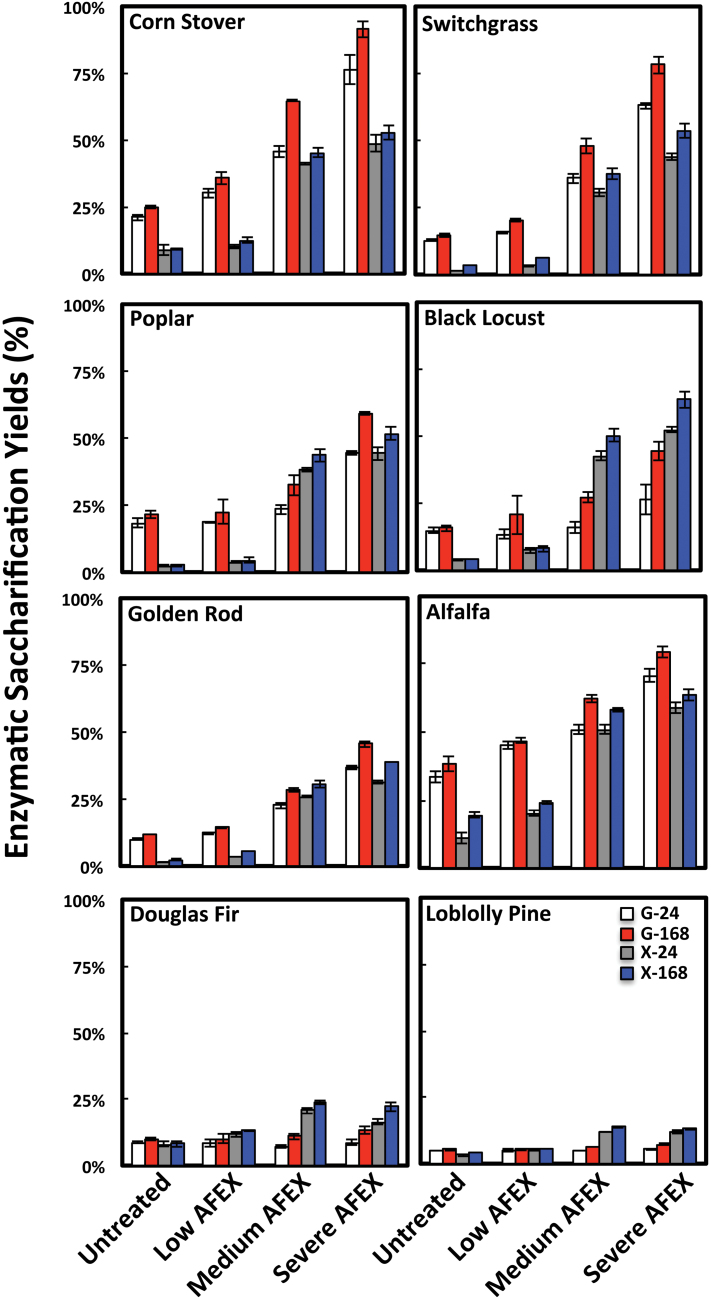
Enzymatic saccharification yields of untreated and AFEX™-pre-treated (for low, medium, and high severity) grasses, dicots, and gymnosperms. Total glucan-to-glucose yields after 24h and 168h are shown in white and red bars, respectively. Total xylan-to-xylose yields after 24h and 168h are shown in grey and blue bars, respectively. Error bars depict standard deviations of data from the mean values reported for assays conducted in triplicate.

The composition of AFEX™-treated biomass has been shown previously to be identical to the composition of untreated samples, since this pre-treatment method is a dry-to-dry process and does not modify the gross cell wall composition (Chundawat *et al.*, 2010, 2011*b*). Furthermore, as shown previously by Wyman and co-workers (Demartini *et al.*, 2011), wet chemistry-based gross cell wall compositional data provide very little information on the ultrastructural modifications within pre-treated plant cell walls that influence its enzymatic deconstruction. Enzymatic saccharification of untreated and AFEX™-pre-treated monocots, woody dicots, herbaceous dicots, and softwoods for 24h and 168h led to a range of glucose (~5–91%) and xylose (~3–64%) yields, as shown in Fig. 7. In all cases, the most severe AFEX™ pre-treatment condition and 168h saccharification time period led to the highest sugar yields compared with the respective untreated samples. The severe AFEX™-pre-treated monocot grasses yielded ~80–90% total available glucan as glucose and 50–55% total available xylan as xylose. The lower hemicellulose conversion for AFEX™-treated samples is not unexpected considering that most commercially available cellulolytic enzyme cocktails (such as C.Tec2 used in this study) lack significant quantities of hemicellulolytic enzymes (Chundawat *et al.*, 2011c). This is further indicated by poor arabinose yield (<20% of the theoretical maximum available; data not shown), which suggests that a significant fraction of the arabinoxylans and/or arabinogalactans in pre-treated monocots and dicots have not been enzymatically hydrolysed. Inclusion of additional hemicellulases has been shown to boost overall hemicellulose conversion further for AFEX™-treated lignocellulosic biomasses (Gao *et al.*, 2010*a*, *b*, 2011). Nevertheless, the overall glucose and xylose yields obtained after prolonged saccharification time periods should be correlated with the relative accessibility of cell wall matrix polymers following AFEX™ pre-treatment. In general, the enzymatic hydrolysis yields for the monocots and hardwoods followed these trends: untreated<low severity AFEX™<<medium severity AFEX™<high severity AFEX™. However, both untreated and pre-treated softwoods yielded the lowest glucose/xylose yields (~10–25%), confirming the significant recalcitrance of these feedstocks to AFEX™ pre-treatment and enzymatic deconstruction compared with the other biomasses tested.

## Conclusion

In this study, biomass/cell wall materials from diverse phylogenetic classes of plants were examined using newly emerging experimental approaches for cell wall analysis. A combination of a recently developed protocol for the pre-treatment of biomass, AFEX™, which is thought to alter wall structure to enhance accessibility to deconstructive enzymes (Chundawat *et al.*, 2011*b*, 2013), and glycome profiling, which is a moderate throughput immunological approach that provides information about wall composition and polymer connectivity (Demartini *et al.*, 2011; Pattathil *et al.*, 2012*a*; Shen *et al.*, 2013; Tan *et al.*, 2013; Zhao *et al.*, 2013), was used for a comparative assessment of overall variations in plant cell wall composition and architecture of native and structurally modified plant cell walls. Overall, the glycome profiling analyses of the diverse plant biomasses analysed in their native form substantiated what was already known about cell wall compositions in terms of the major non-cellulosic glycans that make up these walls. The main focus of these studies was to gain better understanding of cell wall structures/architectures among diverse plant classes based on how the overall non-cellulosic glycan compositions and their extractabilities are affected by AFEX™-induced wall modifications. Further, AFEX™ pre-treatment exerts varying effects on the digestibility of these biomass samples and correlating that digestibility with wall composition is instrumental in making inferences about wall architecture that can be informative about which wall components contribute to recalcitrance among different plant classes. An in-depth understanding of AFEX™-induced modifications to non-cellulosic cell wall components in diverse plant biomass may help to identify those cell wall components that contribute to recalcitrance and thus facilitate the targeting of those components for genetic modification of plant biomass to reduce recalcitrance.

In general, AFEX™ pre-treatments of the angiosperm plant biomasses resulted in a shift of extractable materials from the harsher extraction conditions used in the glycome profiling (e.g. 1M KOH, 4M KOH, and 4M KOHPC) to the less harsh extractives (i.e. oxalate and carbonate). This is particularly evident for corn stover and alfalfa, where the yield of the later extraction steps decreased after AFEX™ pre-treatment, with a corresponding increase in the yields of the earlier extractions (see bar graphs at the top of Figs 1 and 5). The AFEX™ pre-treatment also reduced the recalcitrance of the treated angiosperm biomasses as determined by enhanced sugar release (Fig. 7). AFEX™ pre-treatment had the greatest impact on sugar release from the pre-treated monocot grass and herbaceous dicot walls, with a lower, but still significant improvement in sugar release yields from the woody dicots. The results with the two gymnosperms are in sharp contrast; in general, yields from the glycome profiling extractions were low for both gymnosperms and were little affected by AFEX™ pre-treatment. There was a slight increase in yield in the 4M KOHPC fraction after AFEX™ (see bar graphs on Supplementary Fig. S5 at *JXB* online). Both gymnosperms remained highly recalcitrant to deconstruction after even the most severe AFEX™ treatment. These results suggest fundamental differences in wall architecture between gymnosperms and angiosperms. In particular, gymnosperm walls appear highly resistant to base. The results suggest that removal or destruction of lignin in gymnosperms prior to base pre-treatment might be a more effective pre-treatment than AFEX™ alone. Thus, the very different subunit composition of lignin (almost entirely G for gymnosperms compared with G/S mixed compositions for angiosperms) may be a prime factor in the recalcitrance of gymnosperm walls to pre-treatment and deconstruction.

Glycome profiling of the untreated and AFEX™-treated biomasses allowed some insight into the identity of the polymers affected by AFEX™ and hence into the polymers that might play a key role in connectivities within the walls. One dominant conclusion from these analyses is that there exist different subclasses of each of the major polysaccharides within plant cell walls, and AFEX™ pre-treatment affects the extractability of some of those subclasses, but not others. For example, an enhanced extractability of various subclasses of xylans as indicated by the increased abundance of xylan epitopes in the oxalate and carbonate extracts was observed from pre-treated biomass samples. Similar enhanced extractability was also observed for xyloglucan and pectin/arabinogalactan epitopes in all the angiosperm biomass types as a result of AFEX™ pre-treatments. Interestingly, pre-treated gymnosperm biomasses also showed an enhanced abundance of xylan epitopes in the early extractives (oxalate, carbonate), the amounts of carbohydrate materials recovered in these extracts were very low, and these shifts in extractability appeared to have no effect on sugar release yields from the pre-treated gymnosperm biomass. For AFEX™-pre-treated grasses, enhanced extractability of mixed-linkage glucans, an abundant cell wall component in grasses (Carpita, 1996), was also evident. Previous work has indicated that cell wall hexose/pentose-based oligosaccharides (detected by mass spectrometry) are also readily extracted from AFEX™-pre-treated grasses such as corn stover (Vismeh *et al.*, 2013). These results suggest that AFEX™ pre-treatment results in the loosening of non-cellulosic glycans for angiosperm biomasses. This loosening effect of AFEX™ on plant cell wall matrix glycans could be the net result of a number of factors including: pre-treatment-induced alterations in the overall integrity of cell walls (by disrupting non-covalent interactions between cell wall polymers) or ammonia-catalysed de-esterification (hydrolysis and ammonolysis of lignin–carbohydrate ester linkages and other as yet unidentified base–labile linkages). The consequence of these AFEX™-induced changes in wall structure/architecture is the enhanced access of enzymes to the wall polymers during deconstruction, leading to higher sugar yields from the treated angiosperm biomasses (Fig. 7).

The present studies also demonstrate that different pre-treatment methods modify plant biomass by distinct mechanisms, and studying these pre-treatment-induced cell wall modifications could be informative about variations in plant cell wall structure/architecture and non-cellulosic wall components that contribute to recalcitrance. For instance, it is known that hydrothermal pre-treatment regimes induce hydrolysis and fragmentation of non-cellulosic cell wall glycans, especially for pectic arabinogalactans and lignin-linked glycans (Demartini *et al.*, 2011). Alkaline hydrogen peroxide pre-treatment conditions also appeared to loosen specific subclasses of pectins and hemicelluloses in pre-treated biomass (Li *et al.*, 2014). Previous results from the analyses of switchgrass biomass pre-treated with sugar- and lignin-derived renewable ionic liquids also demonstrated significantly enhanced extractability of hemicelluloses, revealing major effects of ionic liquid-mediated lignin removal on switchgrass cell wall structure/integrity (Socha *et al.*, 2014). Results from the current glycome profiling-based study, employing native and AFEX™-pre-treated pant biomass materials from a wider range of phylogenetic classes of plants, contribute further to the implication of particular subclasses of wall polysaccharides in defining recalcitrant properties of angiosperm cell walls. Overall, these studies demonstrate that monitoring changes in cell wall glycan compositions and their relative extractability following pre-treatment via glycome profiling can assist in obtaining a greater in-depth understanding of plant cell wall structure/architecture and cell wall features that contribute to recalcitrance.

## Supplementary data

Supplementary data are available at *JXB* online.

Figure S1. Heat map analyses of the relative abundance of major non-cellulosic cell wall glycan epitopes in 4M KOH extracts from eight phylogenetically diverse plant biomasses with or without AFEX™ pre-treatment.

Figure S2. Scatter plot analyses of the relative abundance of major non-cellulosic cell wall glycan epitopes in 4M KOH extracts from eight phylogenetically diverse plant biomasses with or without AFEX™ pre-treatment.

Figure S3. Heat map analyses of the relative abundance of major non-cellulosic cell wall glycan epitopes in chlorite extracts from eight phylogenetically diverse plant biomasses with or without AFEX™ pre-treatment.

Figure S4. Scatter plot analyses of the relative abundance of major non-cellulosic cell wall glycan epitopes in chlorite extracts from eight phylogenetically diverse plant biomasses with or without AFEX™ pre-treatment.

Figure S5. Heat map analyses of the relative abundance of major non-cellulosic cell wall glycan epitopes in 4M KOHPC extracts from eight phylogenetically diverse plant biomasses with or without AFEX™ pre-treatment.

Figure S6. Scatter plot analyses of the relative abundance of major non-cellulosic cell wall glycan epitopes in 4M KOHPC extracts from eight phylogenetically diverse plant biomasses with or without AFEX™ pre-treatment.

Figure S7. Cellulose and neutral sugar compositions of the untreated plant biomasses used in this study.

Figure S8. Lignin content, based on the acetyl bromide method, and lignin composition, as S/G/H monomer units, of untreated plant biomasses used in this study.

Table S1. Detailed list of cell wall glycan-directed monoclonal antibodies used for glycome profiling analyses.

Table S2. Analyses of scatter plots generated from normalized monoclonal antibody binding responses derived from glycome profiling data sets of 4M KOH, chlorite, and 4M KOHPC extracts isolated from untreated and medium AFEX^TM^-pre-treated biomass.

Supplementary Data
