# Supplementary material for: Insights into plant cell wall structure, architecture, and integrity using glycome profiling of native and AFEXTM-pre-treated biomass
Source: J Exp Bot. 2015 Apr 23;66(14):4279–94. doi: 10.1093/jxb/erv107 (PMC4493783; doi:10.1093/jxb/erv107)
Supplement: Supplementary Data [file supp_66_14_4279__index.html]

Insights into plant cell wall structure, architecture, and integrity using glycome profiling of native and AFEXTM-pre-treated biomass — Insights into plant cell wall structure, architecture, and integrity using glycome profiling of native and AFEXTM-pre-treated biomass — Supplementary Data 

# Insights into plant cell wall structure, architecture, and integrity using glycome profiling of native and AFEXTM-pre-treated biomass

## Supplementary Data

Data files

**Files in this Data Supplement:**

- Supplementary Data - Supplementary Data
