# Supplementary material for: Insights into plant cell wall structure, architecture, and integrity using glycome profiling of native and AFEXTM-pre-treated biomass
Source: J Exp Bot. 2015 Apr 23;66(14):4279–94. doi: 10.1093/jxb/erv107 (PMC4493783; doi:10.1093/jxb/erv107)
Supplement: Supplementary Data [file supp_erv107_Pattathil_et_al.__2015_JEB_Supplementary_Information_Revised_Manuscript_Final.pdf]

Supplementary Information

Insights into Plant Cell Wall Structure, Architecture and Integrity using Glycome Profiling of Native and AFEX^{TM} Pretreated Biomass

Sivakumar Pattathil*[1,2], Michael G. Hahn[1,2], Bruce E. Dale[3], Shishir P. S. Chundawat*[3,4]

Sivakumar Pattathil
Email : siva@ccrc.uga.edu
Phone: 706 542 4451
Fax : 706 542 4412

Michael G. Hahn
Email: hahn@ccrc.uga.edu

Bruce E. Dale
bdale@egr.msu.edu

Shishir P. S. Chundawat
shishir.chundawat@rutgers.edu
Phone: 848-445-3678
Fax : 517-336-4615

[1]Complex Carbohydrate Research Center, University of Georgia, Athens, GA 30602, USA
[2]BioEnergy Science Center, Oak Ridge National Laboratory, Oak Ridge, TN 37831, USA
[3]DOE Great Lakes Bioenergy Research Center, Department of Chemical Engineering and Materials Science, Michigan State University, East Lansing, MI 48824, USA
[4]Current Address: Department of Chemical and Biochemical Engineering, C-150A Engineering Building, Rutgers The State University of New Jersey, 98 Brett Road, Piscataway, NJ 08854

*Corresponding authors

Supplementary Figure Legends

Supplementary Figure S1: Heat map analyses of the relative abundance of major non-cellulosic cell wall glycan epitopes in 4M KOH extracts from eight phylogenetically diverse plant biomasses with or without AFEX™ pretreatment. 4M KOH extracts were prepared from cell walls isolated from diverse classes of plant biomass as explained in Materials and Methods. The extracts were subsequently screened by ELISA using a comprehensive suite of cell wall glycan directed mAbs. Binding response values are depicted as heat maps with a black-red-bright yellow color scheme, where bright yellow represents strongest binding and black no binding. The dotted boxes outline sets of antibodies whose binding signals were used for the scatter plot analyses shown in Fig. S2. The amount of carbohydrate material recovered per gram of cell wall is depicted in the bar graphs (purple) above the heat maps. The panel on the right hand side of the heat map shows the groups of mAbs based on the class of cell wall glycan they each recognize.

Supplementary Figure S2: Scatter plot analyses of the relative abundance of major non-cellulosic cell wall glycan epitopes in 4M KOH extracts from eight phylogenetically diverse plant biomasses with or without AFEX™ pretreatment. 4M KOH extracts were prepared from cell walls isolated from diverse classes of plant biomass as explained in Materials and Methods. The extracts were subsequently screened by ELISA using a comprehensive suite of cell wall glycan directed mAbs. Comparisons of the relative abundances of epitopes characteristic of three cell wall polysaccharide classes, xyloglucans (blue dots), xylans (red dots) and pectin/arabinogalactans (green dots) in the 4M KOH extracts before and after medium severity AFEX™ pretreatment of diverse plant biomass samples (See Figure 2 for more details). Data are re-plotted from Figure S1, but are normalized to represent mAb binding strength per mass of original cell wall. The red dashed lines denote the expected position if the abundance of these glycan epitopes was unchanged after AFEX™ pretreatment. Data points above and below the dashed lines represent increased or decreased glycan epitope abundance, respectively, after AFEX™ pretreatment. Note that the y-axis scales are different for individual plots to permit visualization of trends and magnitudes of normalized epitope abundances.

Supplementary Figure S3: Heat map analyses of the relative abundance of major non-cellulosic cell wall glycan epitopes in chlorite extracts from eight phylogenetically diverse plant biomasses with or without AFEX™ pretreatment. Chlorite extracts were prepared from cell walls isolated from diverse classes of plant biomass as explained in Materials and Methods. The extracts were subsequently screened by ELISA using a comprehensive suite of cell wall glycan directed mAbs. Binding response values are depicted as heat maps with a black-red-bright yellow color scheme, where bright yellow represents strongest binding and black no binding. The dotted boxes outline sets of antibodies whose binding signals were used for the scatter plot analyses shown in Fig. S4. The amount of carbohydrate material recovered per gram of cell wall is depicted in the bar graphs (purple) above the heat maps. The panel on the right hand side of the heat map shows the groups of mAbs based on the class of cell wall glycan they each recognize.

Supplementary Figure S4: Scatter plot analyses of the relative abundance of major non-cellulosic cell wall glycan epitopes in chlorite extracts from eight phylogenetically diverse plant biomasses with or without AFEX™ pretreatment. Chlorite extracts were prepared from cell walls isolated from diverse classes of plant biomass as explained in Methods. The extracts were subsequently screened by ELISA using a comprehensive suite of cell wall glycan directed mAbs. Comparisons of the relative abundances of epitopes characteristic of three cell wall polysaccharide classes, xyloglucans (blue dots), xylans (red dots) and pectin/arabinogalactans (green dots) in the chlorite extracts before and after medium severity AFEX™ pretreatment of diverse plant biomass samples (See Figure 2 for more details). Data are re-plotted from Figure S3, but are normalized to represent mAb binding strength per mass of original cell wall. The red dashed lines denote the expected position if the abundance of these glycan epitopes was unchanged after AFEX™ pretreatment. Data points above and below the dashed lines represent increased or decreased glycan epitope abundance, respectively, after AFEX™ pretreatment. Note that the y-axis scales are different for individual plots to permit visualization of trends and magnitudes of normalized epitope abundances.

Supplementary Figure S5: Heat map analyses of the relative abundance of major non-cellulosic cell wall glycan epitopes in 4M KOHPC extracts from eight phylogenetically diverse plant biomasses with or without AFEX™ pretreatment. 4M KOHPC extracts were prepared from cell walls isolated from diverse classes of plant biomass as explained in Materials and Methods. The extracts were subsequently screened by ELISA using a comprehensive suite of cell wall glycan directed mAbs. Binding response values are depicted as heat maps with a black-red-bright yellow color scheme, where bright yellow represents strongest binding and black no binding. The dotted boxes outline sets of antibodies whose binding signals were used for the scatter plot analyses shown in Fig. S6. The amount of carbohydrate material recovered per gram of cell wall is depicted in the bar graphs (purple) above the heat maps. The panel on the right hand side of the heat map shows the groups of mAbs based on the class of cell wall glycan they each recognize.

Supplementary Figure S6: Scatter plot analyses of the relative abundance of major non-cellulosic cell wall glycan epitopes in 4M KOHPC extracts from eight phylogenetically diverse plant biomasses with or without AFEX™ pretreatment. 4M KOHPC extracts were prepared from cell walls isolated from diverse classes of plant biomass as explained in Materials and Methods. The extracts were subsequently screened by ELISA using a comprehensive suite of cell wall glycan directed mAbs. Comparisons of the relative abundances of epitopes characteristic of three cell wall polysaccharide classes, xyloglucans (blue dots), xylans (red dots) and pectin/arabinogalactans (green dots) in the 4M KOHPC extracts before and after medium severity AFEX™ pretreatment of diverse plant biomass samples (See Figure 2 for more details). Data are re-plotted from Figure S5, but are normalized to represent mAb binding strength per mass of original cell wall. The red dashed lines denote the expected position if the abundance of these glycan epitopes was unchanged after AFEX™ pretreatment. Data points above and below the dashed lines represent increased or decreased glycan epitope abundance, respectively, after AFEX™

pretreatment. Note that the y-axis scales are different for individual plots to permit visualization of trends and magnitudes of normalized epitope abundances.

Supplementary Figure S7: Cellulose and neutral sugar compositions of the untreated plant biomasses used in this study. Error bars depict standard deviations of data from mean values reported for assays conducted in triplicate. Data plotted in the graph are also shown in the table below to allow comparison between low abundance components.

Supplementary Figure S8: Lignin content, based on the acetyl bromide method (A), and lignin composition, as S/G/H monomer units (B), of untreated plant biomasses used in this study. Error bars depict standard deviations of data from mean values reported for assays conducted in triplicate.

Supplementary Figure S1

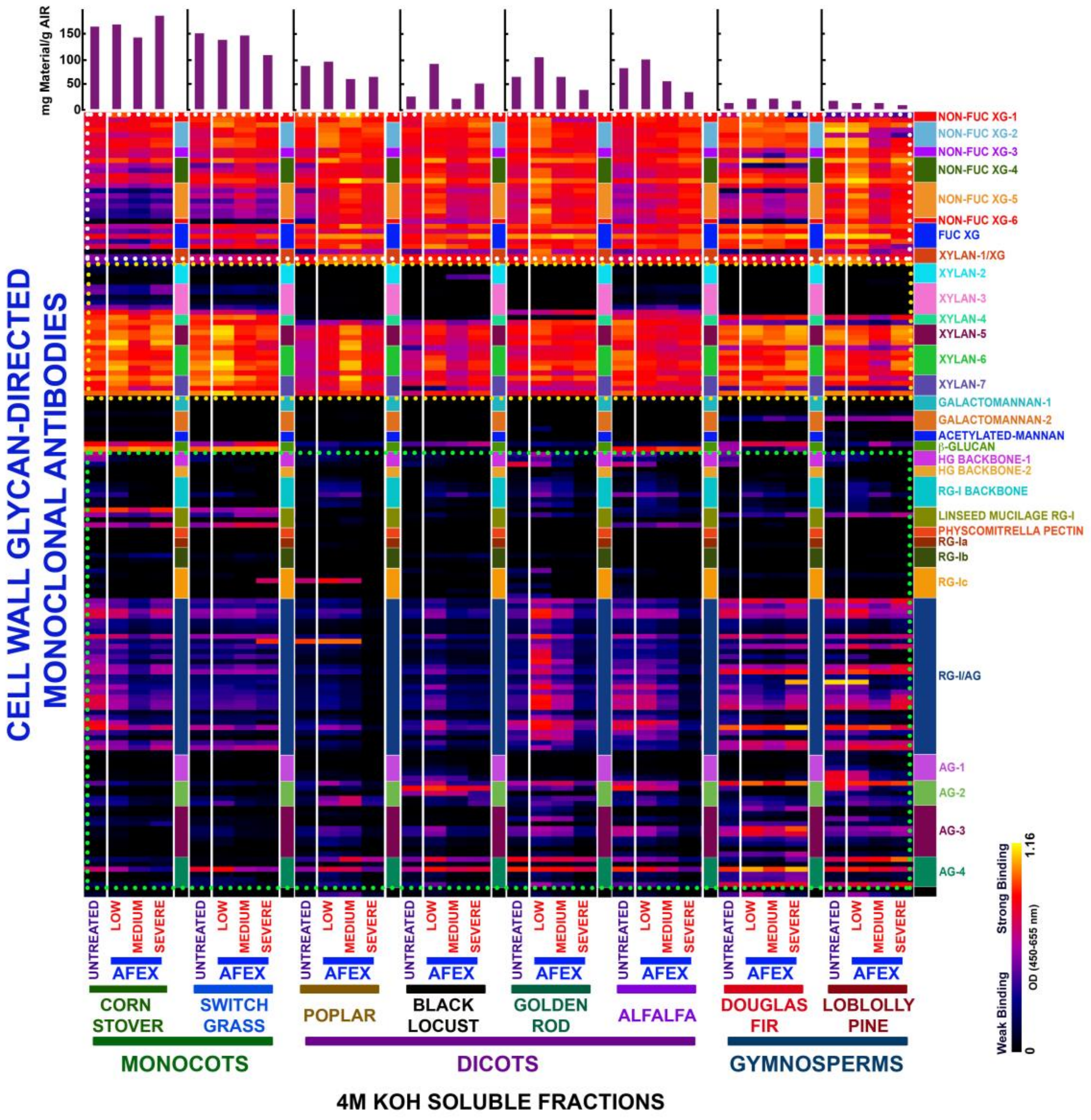

Supplementary Figure S2

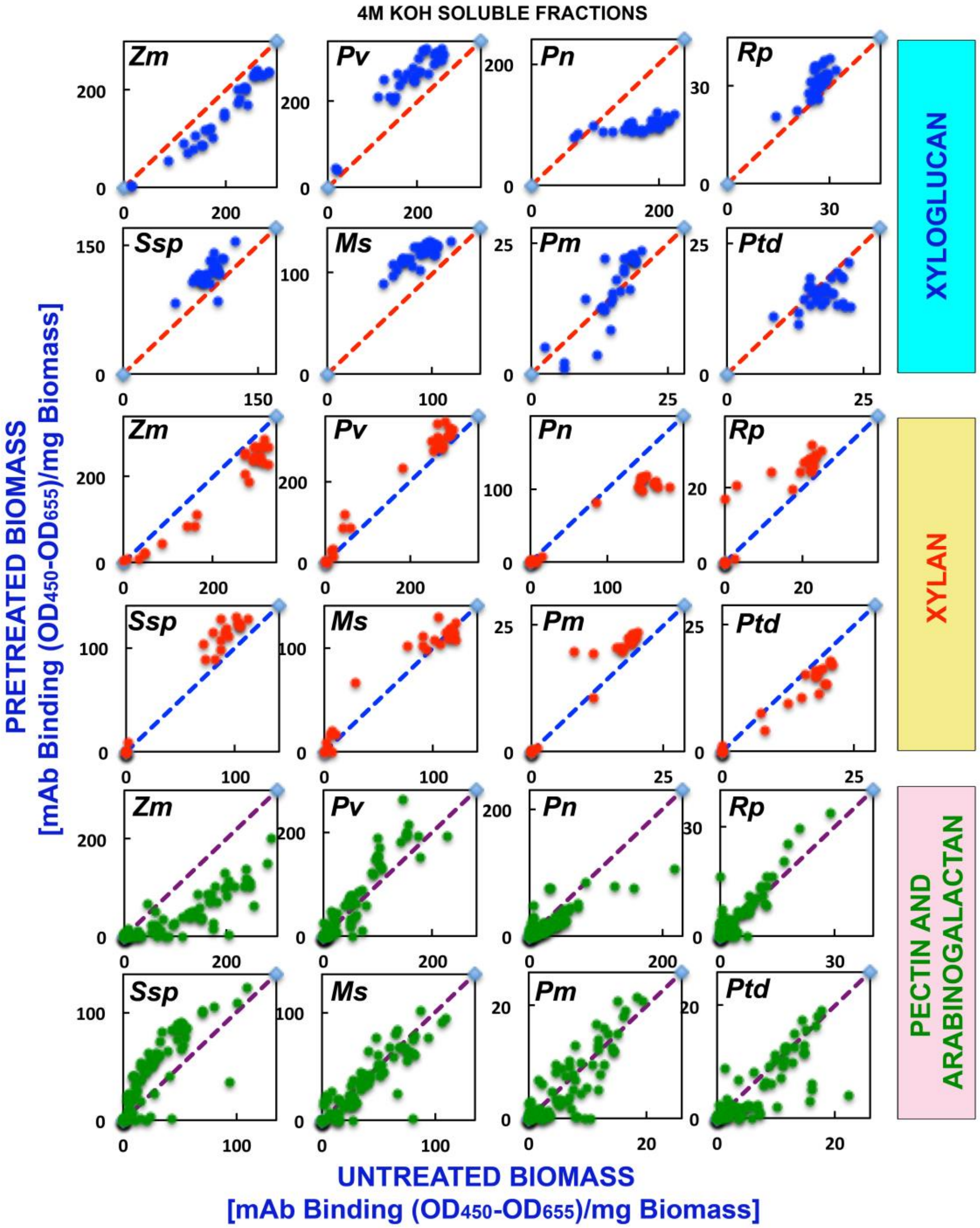

Supplementary Figure S3

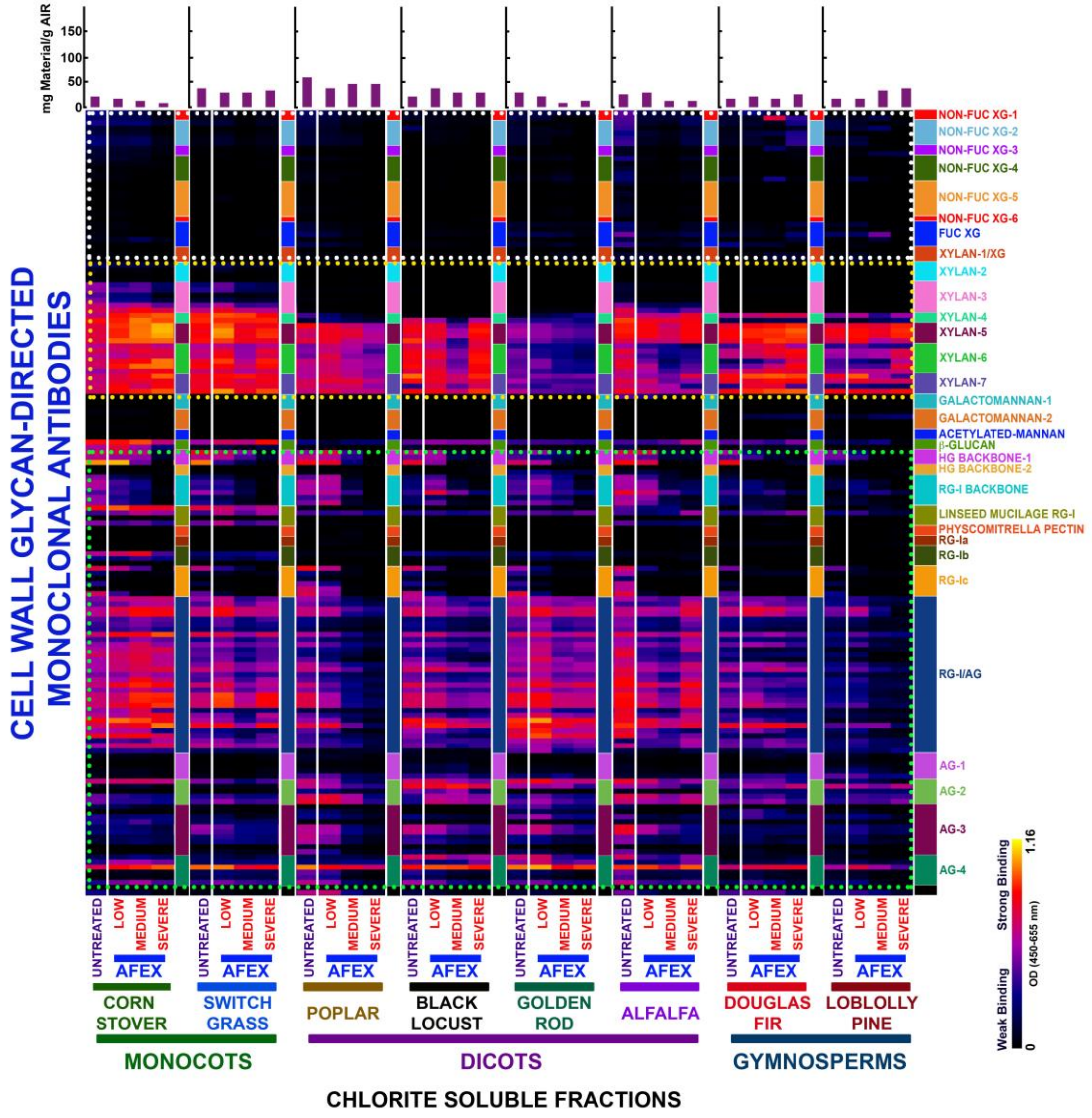

Supplementary Figure S4

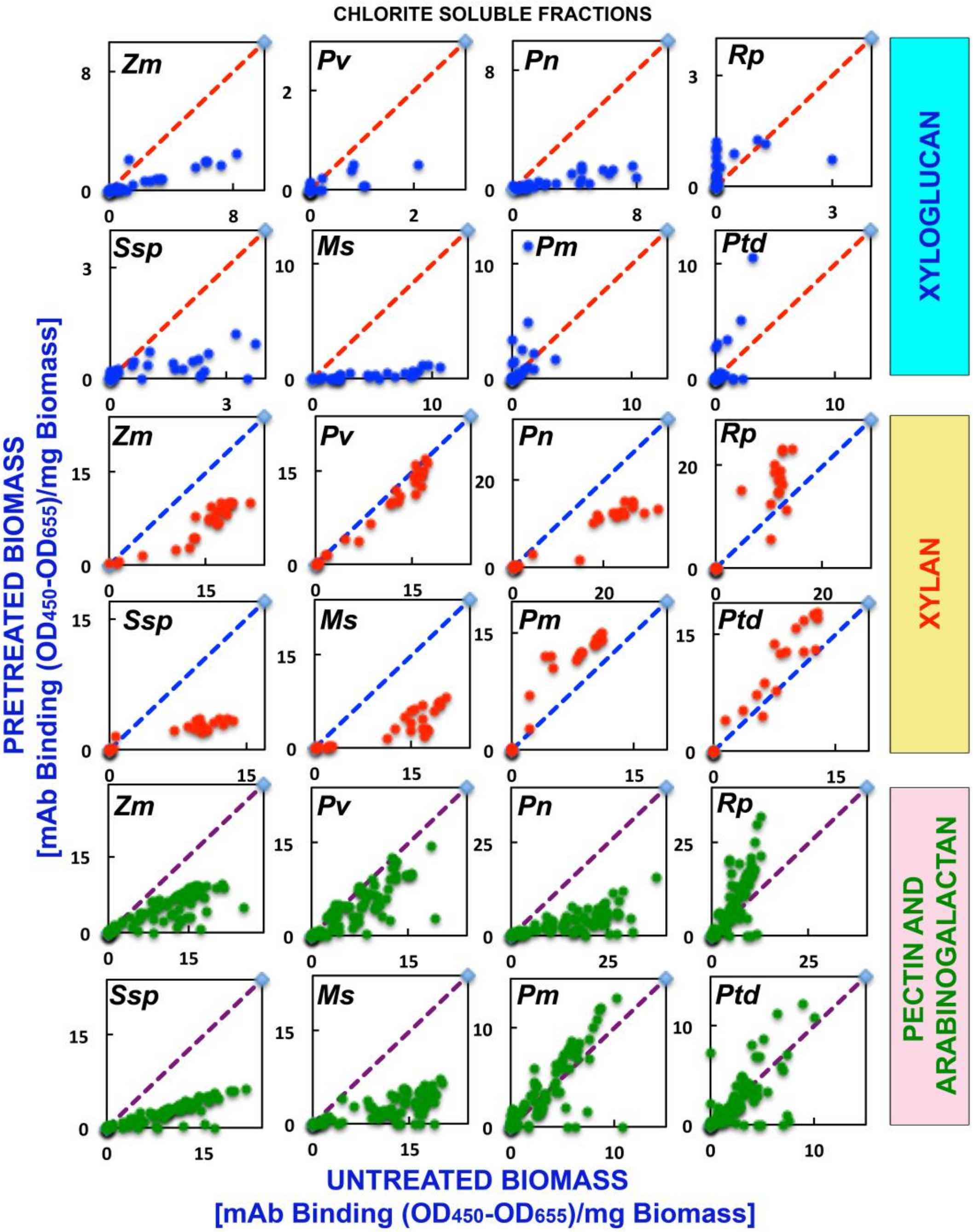

Supplementary Figure S5

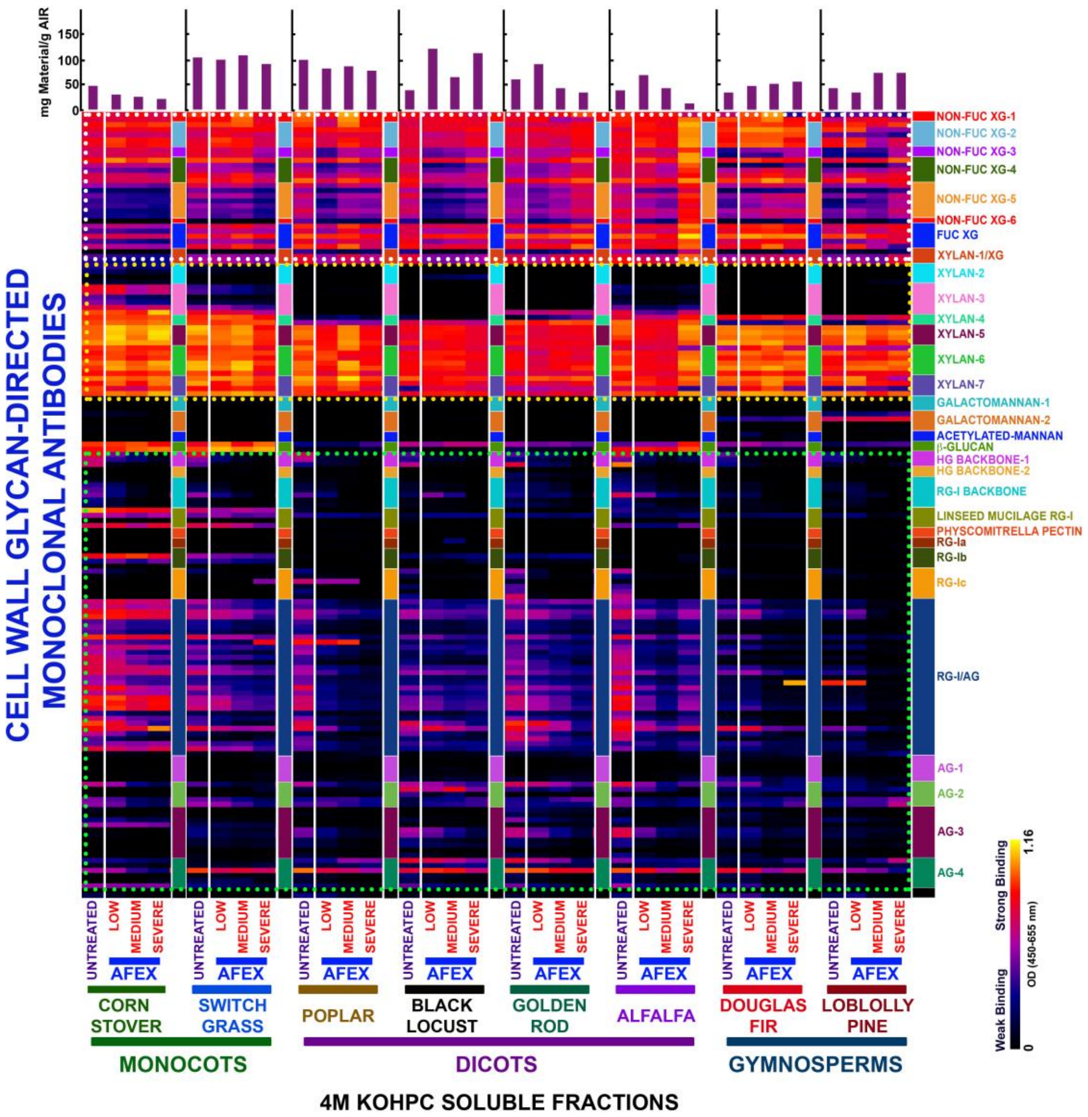

Supplementary Figure S6

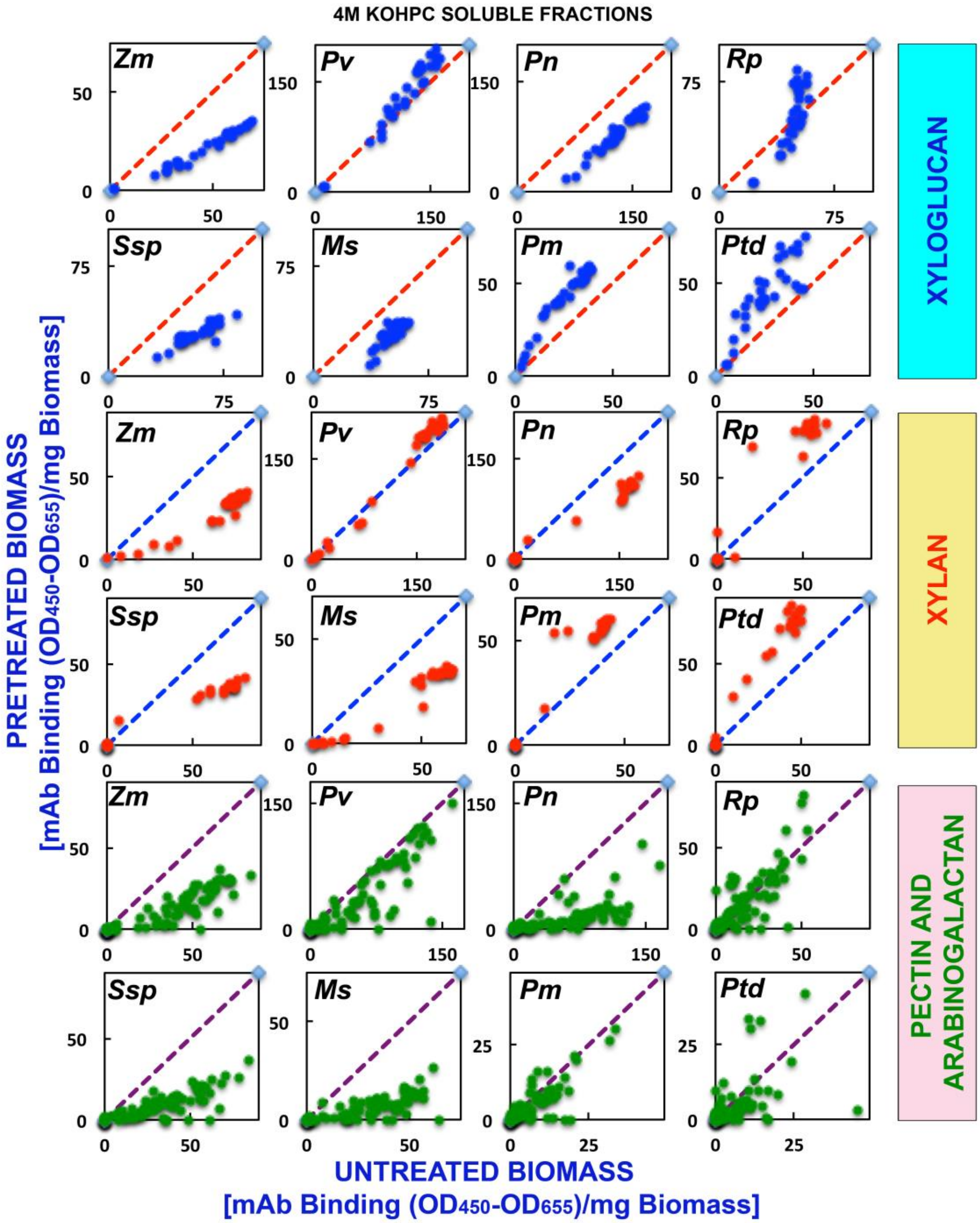

Supplementary Figure S7

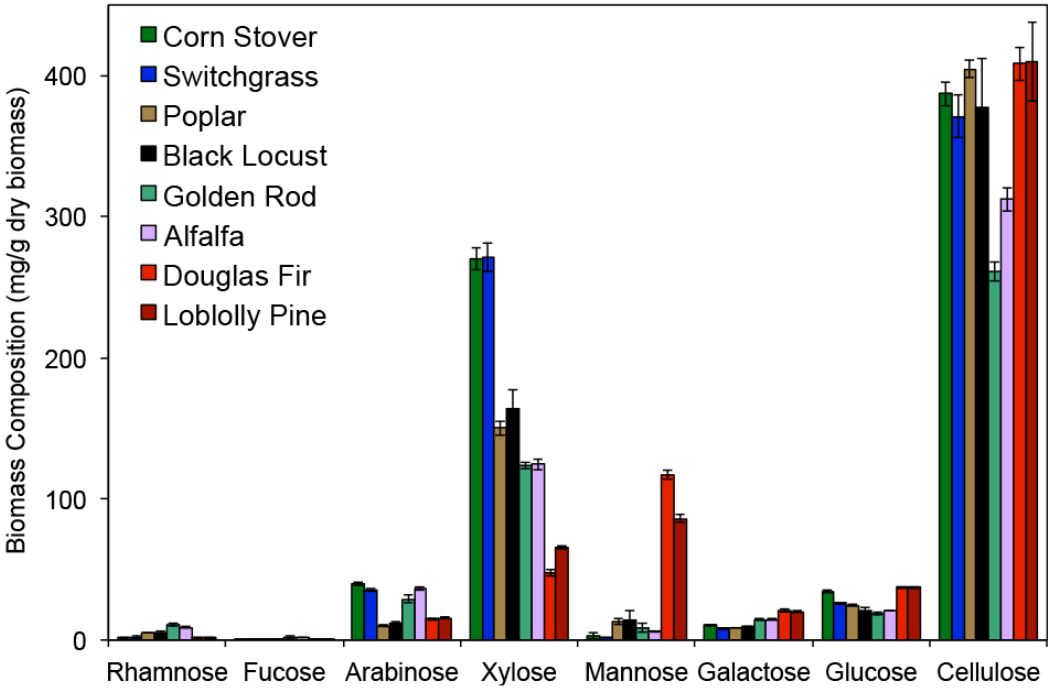

Mean Value

mg/g basis	Rhamnose	Fucose	Arabinose	Xylose	Mannose	Galactose	Glucose	Cellulose
**Corn Stover**	1.7	0.2	40.1	270.1	2.8	10.3	34.5	387.1
**Switchgrass**	1.7	0.4	35.3	271.0	1.7	8.2	26.1	371.1
**Poplar**	5.0	0.6	9.9	150.0	13.3	8.7	24.7	404.6
**Black Locust**	4.7	1.0	11.7	163.9	13.9	9.1	21.2	377.1
**Golden Rod**	11.2	1.5	29.1	123.5	8.8	14.6	18.6	261.3
**Alfalfa**	9.0	1.9	36.1	124.4	6.2	14.6	20.6	312.3
**Douglas Fir**	1.4	0.2	14.7	47.6	117.2	21.1	37.3	408.3
**Loblolly Pine**	1.4	0.2	15.6	65.7	86.1	20.0	37.3	409.6

Standard Deviation

	Rhamnose	Fucose	Arabinose	Xylose	Mannose	Galactose	Glucose	Cellulose
**Corn Stover**	0.7	0.3	1.4	7.6	2.2	0.4	1.0	8.5
**Switchgrass**	1.5	0.4	0.7	9.8	0.3	0.5	0.7	15.1
**Poplar**	0.1	0.0	0.5	4.9	2.2	0.0	1.1	6.0
**Black Locust**	1.1	0.2	0.8	13.3	6.4	0.2	1.4	35.3
**Golden Rod**	1.2	1.3	3.0	2.2	3.2	1.0	0.8	6.9
**Alfalfa**	0.1	0.0	1.1	3.7	0.5	0.2	0.3	8.3
**Douglas Fir**	0.9	0.4	0.6	2.6	3.3	1.1	0.6	11.7
**Loblolly Pine**	0.7	0.4	0.4	1.1	3.1	0.7	0.7	27.9

Supplementary Figure S8

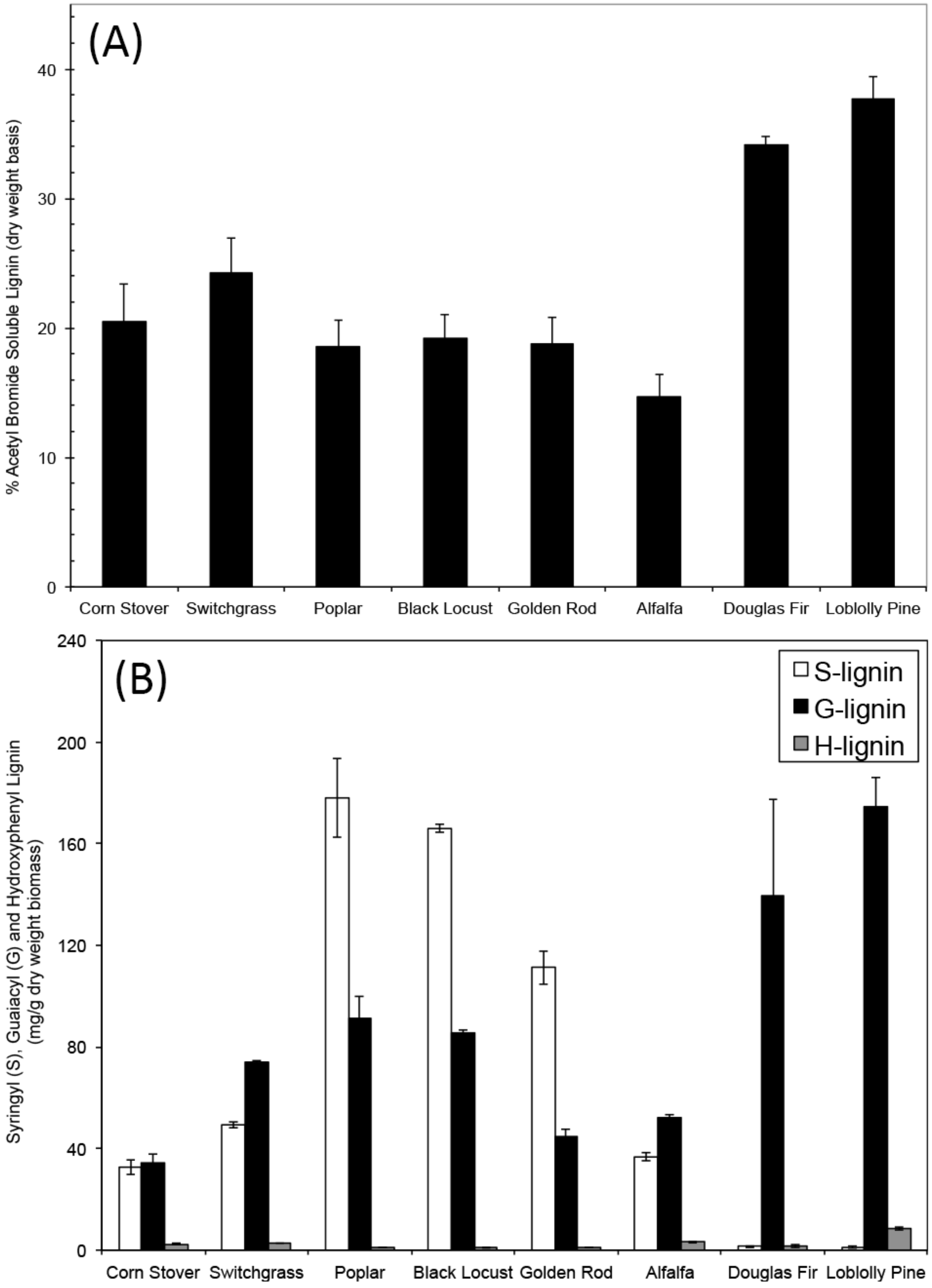

Supplementary Tables

Supplementary Table S1: Detailed list of cell wall glycan-directed monoclonal antibodies (mAbs) used for glycome profiling analyses. The groupings of antibodies are based on a hierarchical clustering of ELISA data generated from a screen of all mAbs against a comprehensive panel of plant polysaccharide preparations (Pattathil et al., 2010; Pattathil et al., 2012) that clusters mAbs according to the predominant polysaccharides that they recognize. The majority of listings link to the Wall*Mab*DB plant cell wall monoclonal antibody database (http://www.wallmabdb.net) that provides detailed descriptions of each mAb, including immunogen, antibody isotype, epitope structure (to the extent known), supplier information, and related literature citations.

Glycan Group Recognized	mAb Names
Non-Fucosylated Xyloglucan-1	CCRC-M95<br>CCRC-M101
Non-Fucosylated Xyloglucan-2	CCRC-M104<br>CCRC-M89<br>CCRC-M93<br>CCRC-M87<br>CCRC-M88
Non-Fucosylated Xyloglucan-3	CCRC-M100<br>CCRC-M103
Non-Fucosylated Xyloglucan-4	CCRC-M58<br>CCRC-M86<br>CCRC-M55<br>CCRC-M52<br>CCRC-M99

Non-Fucosylated Xyloglucan-5	CCRC-M54<br>CCRC-M48<br>CCRC-M49<br>CCRC-M96<br>CCRC-M50<br>CCRC-M51<br>CCRC-M53
Non-Fucosylated Xyloglucan-6	CCRC-M57
Fucosylated Xyloglucan	CCRC-M102<br>CCRC-M39<br>CCRC-M106<br>CCRC-M84<br>CCRC-M1
Xylan-1/XG	CCRC-M111<br>CCRC-M108<br>CCRC-M109
Xylan-2	CCRC-M119<br>CCRC-M115<br>CCRC-M110<br>CCRC-M105
Xylan-3	CCRC-M117<br>CCRC-M113<br>CCRC-M120<br>CCRC-M118<br>CCRC-M116<br>CCRC-M114
Xylan-4	CCRC-M154<br>CCRC-M150

Xylan-5	CCRC-M144<br>CCRC-M146<br>CCRC-M145<br>CCRC-M155
Xylan-6	CCRC-M153<br>CCRC-M151<br>CCRC-M148<br>CCRC-M140<br>CCRC-M139<br>CCRC-M138
Xylan-7	CCRC-M160<br>CCRC-M137<br>CCRC-M152<br>CCRC-M149
Galactomannan-1	CCRC-M75<br>CCRC-M70<br>CCRC-M74
Galactomannan-2	CCRC-M166<br>CCRC-M168<br>CCRC-M174<br>CCRC-M175
Acetylated Mannan	CCRC-M169<br>CCRC-M170
β-Glucan	LAMP<br>BG1
HG Backbone-1	CCRC-M131<br>CCRC-M38<br>JIM5

HG Backbone-2	JIM136<br>JIM7
RG-I Backbone	CCRC-M69<br>CCRC-M35<br>CCRC-M36<br>CCRC-M14<br>CCRC-M129<br>CCRC-M72
Linseed Mucilage RG-I	JIM3<br>CCRC-M40<br>CCRC-M161<br>CCRC-M164
Physcomitrella Pectin	CCRC-M98<br>CCRC-M94
RG-Ia	CCRC-M5<br>CCRC-M2
RG-Ib	JIM137<br>JIM101<br>CCRC-M61<br>CCRC-M30
RG-Ic	CCRC-M23<br>CCRC-M17<br>CCRC-M19<br>CCRC-M18<br>CCRC-M56<br>CCRC-M16
RG-I/Arabinogalactan	CCRC-M60<br>CCRC-M41<br>CCRC-M80<br>CCRC-M79

CCRC-M44
CCRC-M33
CCRC-M32
CCRC-M13
CCRC-M42
CCRC-M24
CCRC-M12
CCRC-M7
CCRC-M77
CCRC-M25
CCRC-M9
CCRC-M128
CCRC-M126
CCRC-M134
CCRC-M125
CCRC-M123
CCRC-M122
CCRC-M121
CCRC-M112
CCRC-M21
JIM131
CCRC-M22
JIM132
JIM1
CCRC-M15
CCRC-M8
JIM16

Arabinogalactan-1

JIM93
JIM94
JIM11
MAC204
JIM20

Arabinogalactan-2	JIM14
	JIM19
	JIM12
	CCRC-M133
	CCRC-M107
Arabinogalactan-3	JIM4
	CCRC-M31
	JIM17
	CCRC-M26
	JIM15
	JIM8
	CCRC-M85
	CCRC-M81
	MAC266
	PN 16.4B4
Arabinogalactan-4	MAC207
	JIM133
	JIM13
	CCRC-M92
	CCRC-M91
	CCRC-M78
Unidentified	MAC265
	CCRC-M97

Supplementary Table S2: Analyses of scatter plots generated from normalized mAb binding responses derived from glycome profiling data sets of 4M KOH, chlorite and 4M KOHPC extracts isolated from untreated and medium AFEX^{TM} pretreated biomass (Figures, S2, S4 and S6). The table summarizes overall status in the abundance of epitopes recognized by XG, xylan and pectin/arabinogalactan mAbs in each extract from medium regime AFEX™ pretreated biomass as "reduced", "enhanced" or "no change" in comparison to the respective untreated biomass. Green and grey highlights denote similar and non-similar trends in related phylogenetic classes of plant biomass, respectively.

Biomass Type	Xyloglucan Epitope Abundance			Xylan Epitope Abundance			Pectin/AG Epitope Abundance		
	4M KOH	Chlorite	4M KOHPC	4M KOH	Chlorite	4M KOHPC	4M KOH	Chlorite	4M KOHPC
Overall scatterplot trends exhibited by AFEX™ pretreated Angiosperm Monocot Grasses									
Corn Stover	Reduced	Reduced	Reduced	Reduced	Reduced	Reduced	Reduced	Reduced	Reduced
Switchgrass	Enhanced	Reduced	No change	Enhanced	No change	No change	No change	Reduced	Reduced
Overall scatterplot trends exhibited by AFEX™ pretreated Woody Angiosperm Dicots									
Poplar	Reduced	Reduced	Reduced	Reduced	Reduced	Reduced	No change	Reduced	Reduced
Black Locust	Enhanced	Enhanced	No change	Enhanced	Enhanced	Enhanced	No change	Enhanced	No change
Overall scatterplot trends exhibited by AFEX™ pretreated Herbaceous Angiosperm Dicots									
Golden Rod	Enhanced	Reduced	Reduced	Enhanced	Reduced	Reduced	Enhanced	Reduced	Reduced
Alfalfa	Enhanced	Reduced	Reduced	No change	Reduced	Reduced	No change	Reduced	Reduced
Overall scatterplot trends exhibited by AFEX™ pretreated Woody Gymnosperms									
Douglas Fir	No change	No change	Enhanced	No change	Enhanced	Enhanced	No change	No change	No change
Loblolly Pine	No change	No change	Enhanced	No change	Enhanced	Enhanced	No change	No change	No change

References:

Pattathil S, Avci U, Baldwin D, *et al.* 2010. A comprehensive toolkit of plant cell wall glycan-directed monoclonal antibodies. *Plant Physiology* **153,** 514-525.

Pattathil S, Avci U, Miller JS, Hahn MG. 2012. Immunological approaches to plant cell wall and biomass characterization: Glycome profiling. In: Himmel M (ed) Biomass Conversion: Methods and Protocols. Springer Science + Business Media, LLC, New York, NY, pp 61-72.
